# Exposures and Emissions in Coffee Roasting Facilities and Cafés: Diacetyl, 2,3-Pentanedione, and Other Volatile Organic Compounds

**DOI:** 10.3389/fpubh.2020.561740

**Published:** 2020-09-18

**Authors:** Ryan F. LeBouf, Brie Hawley Blackley, Alyson R. Fortner, Marcia Stanton, Stephen B. Martin, Caroline P. Groth, Tia L. McClelland, Matthew G. Duling, Dru A. Burns, Anand Ranpara, Nicole Edwards, Kathleen B. Fedan, Rachel L. Bailey, Kristin J. Cummings, Randall J. Nett, Jean M. Cox-Ganser, M. Abbas Virji

**Affiliations:** ^1^Respiratory Health Division, National Institute for Occupational Safety and Health, Morgantown, WV, United States; ^2^Department of Biostatistics, School of Public Health, West Virginia University, Morgantown, WV, United States; ^3^California Department of Public Health, Richmond, CA, United States

**Keywords:** coffee roasting and packaging, cafe, exposure assessment, volatile organic compounds, diacetyl, 2,3-pentanedione (acetyl propionyl)

## Abstract

Roasted coffee and many coffee flavorings emit volatile organic compounds (VOCs) including diacetyl and 2,3-pentanedione. Exposures to VOCs during roasting, packaging, grinding, and flavoring coffee can negatively impact the respiratory health of workers. Inhalational exposures to diacetyl and 2,3-pentanedione can cause obliterative bronchiolitis. This study summarizes exposures to and emissions of VOCs in 17 coffee roasting and packaging facilities that included 10 cafés. We collected 415 personal and 760 area full-shift, and 606 personal task-based air samples for diacetyl, 2,3-pentanedione, 2,3-hexanedione, and acetoin using silica gel tubes. We also collected 296 instantaneous activity and 312 instantaneous source air measurements for 18 VOCs using evacuated canisters. The highest personal full-shift exposure in part per billion (ppb) to diacetyl [geometric mean (GM) 21 ppb; 95th percentile (P95) 79 ppb] and 2,3-pentanedione (GM 15 ppb; P95 52 ppb) were measured for production workers in flavored coffee production areas. These workers also had the highest percentage of measurements above the NIOSH Recommended Exposure Limit (REL) for diacetyl (95%) and 2,3-pentanedione (77%). Personal exposures to diacetyl (GM 0.9 ppb; P95 6.0 ppb) and 2,3-pentanedione (GM 0.7 ppb; P95 4.4 ppb) were the lowest for non-production workers of facilities that did not flavor coffee. Job groups with the highest personal full-shift exposures to diacetyl and 2,3-pentanedione were flavoring workers (GM 34 and 38 ppb), packaging workers (GM 27 and 19 ppb) and grinder operator (GM 26 and 22 ppb), respectively, in flavored coffee facilities, and packaging workers (GM 8.0 and 4.4 ppb) and production workers (GM 6.3 and 4.6 ppb) in non-flavored coffee facilities. Baristas in cafés had mean full-shift exposures below the RELs (GM 4.1 ppb diacetyl; GM 4.6 ppb 2,3-pentanedione). The tasks, activities, and sources associated with flavoring in flavored coffee facilities and grinding in non-flavored coffee facilities, had some of the highest GM and P95 estimates for both diacetyl and 2,3-pentanedione. Controlling emissions at grinding machines and flavoring areas and isolating higher exposure areas (e.g., flavoring, grinding, and packaging areas) from the main production space and from administrative or non-production spaces is essential for maintaining exposure control.

## Introduction

The worldwide demand for roasted coffee and coffee beverages is on the rise. Coffee consumption in the United States increased from 1.43 billion kilograms (kg) in 2013/2014 to 1.55 billion in 2017/2018 (1). The United States is forecast to be the second-largest importer of coffee beans (1.57 billion kg) behind the European Union (2.88 billion kg) in 2019/2020 (2). In 2016, the US coffee industry (NAICS 311920) had 15,911 full-time and part-time employees (3) with 11% in small-sized (<20 employees) businesses representing 73% of establishments, 37% in medium-sized (≥20 to <500 employees) businesses representing 7% of establishments, and 52% in large-sized (500+ employees) businesses representing 20% of establishments (4).

Roasted coffee production and café workers can be exposed to a variety of chemicals at work. Roasted coffee emits carbon monoxide (CO), carbon dioxide (CO_2_), and a wide range of VOCs (5–10). Emitted VOCs include alpha-diketones such as 2,3-butanedione (diacetyl), 2,3-pentanedione (acetyl propionyl), and 2,3-hexanedione. Grinding roasted coffee beans produces a greater surface area for off-gassing (sometimes called degassing) of CO, CO_2_, and VOCs (11, 12). In addition to occurring naturally in roasted coffee, diacetyl and 2,3-pentanedione are added as ingredients in food flavorings used in some food products, including ground or whole bean coffee to make flavored coffee (13–15). Acetoin and 2,3-pentanedione are common substitutes for diacetyl in flavorings (16).

The National Institute for Occupational Safety and Health (NIOSH) has published full-shift Recommended Exposure Limits (RELs) of 5.0 parts per billion (ppb) for diacetyl and 9.3 ppb for 2,3-pentanedione. The NIOSH short-term exposure limits (STELs) are 25 ppb for diacetyl and 31 ppb for 2,3-pentanedione averaged over a 15 min time period. Short-term peak exposures might be relevant for respiratory health, particularly when tasks are repeated multiple times per day.

The NIOSH objective in establishing RELs for diacetyl and 2,3-pentanedione is to reduce the risk of respiratory impairment (decreased lung function) and the severe irreversible lung disease obliterative bronchiolitis associated with occupational exposure to these chemicals. These exposure limits were derived from a risk assessment of flavoring-exposed workers. At an exposure equal to the diacetyl REL, the risk of adverse health effects is low. NIOSH estimated about 1 in 1,000 workers exposed to diacetyl levels of 5 ppb as a time-weighted average (TWA) for 8 h a day, 40 h a week for a 45-year working lifetime would develop reduced lung function (defined as forced expiratory volume in 1 s (FEV1) below the lower limit of normal) as a result of that exposure. NIOSH predicted that around 1 in 10,000 workers exposed to diacetyl at 5 ppb for a 45-year working lifetime would develop more severe lung function reduction [FEV1 below 60% predicted, defined as at least moderately severe by the American Thoracic Society (17)]. Workers exposed for less time would be at lower risk for adverse lung effects. NIOSH RELs should be used as a guideline to indicate when exposure reduction steps should be taken in the workplace. The American Conference of Governmental Industrial Hygienists (ACGIH^®^) has a threshold limit value (TLV^®^) for diacetyl of 10 ppb, as a full-shift time-weighted average exposure and a STEL of 20 ppb. Diacetyl is on the 2020 ACGIH TLV list of chemicals under study. ACGIH does not have a TLV^®^-TWA or a STEL for 2,3-pentanedione. Occupational exposure limits for 2,3-hexanedione and acetoin do not exist.

Inhalational exposure to diacetyl has been associated with a lung disease called obliterative bronchiolitis (18). Obliterative bronchiolitis is a severe, often disabling, lung disease that involves scarring of the very small airways (i.e., bronchioles). Symptoms of this disease may include cough, shortness of breath on exertion, or wheeze, and do not typically improve away from work (19). Occupational obliterative bronchiolitis has been identified in flavoring manufacturing workers and microwave popcorn workers who worked with flavoring chemicals or butter flavorings (14, 20, 21). A diacetyl substitute, 2,3-Pentanedione, was found to have respiratory toxicity in animal studies similar to that of diacetyl (22, 23). In one animal study, there was evidence that 2,3-hexanedione might also damage the lungs, but it appeared to be less toxic than diacetyl and 2,3-pentanedione (24). Obliterative bronchiolitis has been reported among workers at two coffee roasting and packaging facilities that produced both unflavored and flavored coffee (13, 25, 26). At one of those facilities, all former workers diagnosed with obliterative bronchiolitis had worked in the flavoring area (13). Current workers at that facility had excess shortness of breath and obstruction on spirometry, consistent with undiagnosed lung disease. Respiratory morbidity among current workers was associated with working in areas where coffee was flavored, and areas where grinding and packaging of unflavored coffee occurred (13). However, to our knowledge, no cases of obliterative bronchiolitis have been reported in workers at coffee roasting and packaging facilities that produce only unflavored coffee.

In an effort to characterize occupational exposures to alpha-diketones and other VOCs, we performed exposure assessments at 17 coffee facilities, some of which included cafés, through the NIOSH Health Hazard Evaluation (HHE) program. The HHE program responds to requests to investigate exposure or health issues in workplaces from employers, employees, or union representatives. One HHE request was from employees and 16 were from employers. The respiratory abnormalities of the workforce at these 17 facilities included nose and eye symptoms, wheeze, and rare abnormal spirometry (5%), and is described in detail elsewhere (27).

## Materials and Methods

### Facility Characteristics

Annual roasted coffee production at the 17 facilities ranged from 14,000 to 4,080,000 kgs per year (Table 1). The median number of production workers was seven (range: 3–100). The majority of facilities produced unflavored whole-bean coffee. Four of 17 facilities flavored coffee during the survey; one facility flavored ground coffee and three facilities flavored whole-bean and then ground the flavored beans. One facility flavored coffee on occasion but did not do so during the survey. Eight facilities had either one onsite or one offsite café; one facility had two offsite cafés. Facilities were sampled between July 2015 and September 2017 during a variety of seasons and in a number of geographical locations, which influenced the temperature during sampling and amount of natural ventilation occurring from open doors or windows.

**Table 1:** Production characteristics of 17 sampled coffee facilities.

**Facility**	**Production area (m^**2**^)**	**# Production/total workers**	**Annual production roasted coffee (tons/year)**	**Percentage whole bean coffee (%)**	**Flavoring during survey (yes/no)**	**Café**	**Season during sampling**	**US Climate region**
1	1.0 × 10^2^	4/4	1.6 × 10^1^	90	No	Offsite^a^	Spring	Northeast
2	7.4 × 10^1^	3/6	2.0 × 10^1^	45	Yes	–	Winter	Ohio Valley
3	9.3 × 10^1^	10/19	3.0 × 10^1^	70	No	Offsite	Winter	Northwest
4	2.0 × 10^2^	3/6	3.9 × 10^1^	95	Yes	–	Winter	Southwest
5	2.3 × 10^2^	9/18	4.5 × 10^1^	97	No^b^	Offsite^c^	Fall	Southeast
6	1.1 × 10^2^	4/5	6.0 × 10^1^	75	No	–	Spring	Southeast
7	9.3 × 10^1^	3/9	6.0 × 10^1^	75	No	–	Spring	Southeast
8	4.0 × 10^2^	6/20	9.6 × 10^1^	97	No	Onsite	Spring	Upper Midwest
9	1.0 × 10^3^	13/26	1.3 × 10^2^	95	No	–	Summer/Spring	Upper Midwest
10	2.3 × 10^2^	7/19	1.4 × 10^2^	90	No	Onsite	Summer	Upper Midwest
11	2.9 × 10^2^	5/10	1.6 × 10^2^	97	No	Offsite	Spring	Upper Midwest
12	6.5 × 10^2^	10/49	1.7 × 10^2^	75	No	Onsite	Winter	Upper Midwest
13	9.3 × 10^2^	11/43	2.5 × 10^2^	65	No	Offsite	Spring	Upper Midwest
14	2.1 × 10^3^	6/54	1.4 × 10^3^	35	Yes	–	Summer	Upper Midwest
15	4.2 × 10^3^	20/90	2.6 × 10^3^	73	No	–	Spring	Northeast
16	4.9 × 10^3^	100/120	3.5 × 10^3^	40	Yes	–	Summer	Upper Midwest
17	4.5 × 10^3^	50/150	4.5 × 10^3^	60	No	Onsite	Fall	Southwest

a *Two locations*.

b *Facility does flavor coffee but did not during survey*.

c *Not sampled*.

“–,” *Café not present*.

### Process and Task Description

The main steps in roasting and packaging coffee are typically: (1) receiving green (raw) beans, (2) roasting green beans, (3) grinding roasted beans, (4) weighing and packaging roasted and ground coffee, and (5) shipping. Some facilities also flavored roasted ground or whole bean coffee with liquid flavoring before packaging or grinding.

Green beans were received in jute or burlap bags from countries around the world and stored in designated areas or in the main production space. Workers moved bags of green beans on pallets using a forklift or carried bags to a storage area. The first step in the production process was weighing and transferring the green beans to a conduction or convection roaster. Some facilities pneumatically fed green beans into the roasters. Some facilities blended green coffee beans before roasting and others blended roasted beans after roasting. A roaster operator monitored roasting time and temperature that depended on the green bean origin and desired roast level (e.g., light, medium, dark). Occasionally, the roaster operator would pull a sample of beans from the roaster to check the color and smell of the beans. In a majority of facilities (16 of 17), the roasted coffee beans were sent to downdraft or updraft cooling drums and mixed by an agitator to accelerate cooling. Cooling systems exhausted out through the roof or side of the buildings. After roasting, the roasted coffee beans were sent through a destoner (to remove any foreign objects) and transferred to containers or silos. In some facilities, the roasted product was allowed to off-gas in a bin or silo located in a designated roasted bean storage area for 12 to 48 h if the product was to be packaged in a bag without a one-way valve. At three of the four facilities that flavored coffee, the flavoring and cooled roasted coffee were measured and added to a bucket with a lid or a plastic bag that was sealed. The worker then shook the flavored coffee container by hand. At the fourth facility, a dedicated flavoring room received whole beans through a pneumatic system. The flavoring room attendant manually mixed the liquid flavorings in an 18-kg pail, then poured it into an automatic ribbon blender, which mixed the flavorings with the whole beans or ground coffee. Some coffee was ground before packaging. Grinders were manual 0.45-kg (1-lb) to 2.3-kg (5-lb) machines or automated machines capable of grinding up to 318 kg per hour. Whole-bean and ground coffee were manually packaged into bags (with and without one-way valves) or other containers, or automatically packaged using weighing and packaging lines. These lines were monitored to assure quality of packaging. In the event of packaging defects, some re-work of product was required. Re-work involved manually cutting open defective packaging and returning coffee to a packaging line. Bags were generally heat-sealed to complete the finished product.

During the production process, companies tested green and roasted beans to ensure quality. The facilities had quality control areas where roasted beans and brews were prepared and assessed. Upon receipt, a worker profiled the green beans to determine the best roast temperature and time. Green beans stored in silos were monitored over time as they aged and roasting specifications were adjusted to account for any changes in the green beans. Within each specific type of roast, the beans were generally packaged in the order they were roasted to ensure freshness.

Various cleaning techniques were used throughout the production areas. Workers used brooms to sweep the production floor, wet or dry wipes on tabletops and equipment surfaces, and compressed air to remove coffee bean dust from surfaces and equipment. In some facilities, maintenance workers maintained and repaired production equipment and customers' coffee roasting equipment (roasters, grinders, and espresso machines) as needed.

Tasks performed by workers during the production process included miscellaneous production (e.g., moving, loading, or scooping green beans; making labels; and moving pallets of coffee), roasting coffee beans, pulling samples of beans during roasting, quality control, moving roasted beans or ground coffee (e.g., scooping roasted whole bean coffee into packaging machine, pouring whole beans into buckets to hand blend, pouring beans into storage bins, etc.), grinding coffee beans, flavoring coffee, packaging coffee, packaging rework, cleaning machines, maintenance of machines, and miscellaneous café tasks (Supplementary Table 1). Suspected sources of emissions included roasting, roasted coffee, roasted coffee in bag, roasted coffee in container, roaster cooling drum, roaster door, sampler roaster, QC grinder, miscellaneous QC, ground coffee, heat sealing bags, packaging roasted coffee, flavoring, flavored coffee, café grinder, and miscellaneous café (Supplementary Table 2).

Workers were not required to wear company uniforms or protective clothing. We did not observe workers wearing respiratory protection for chemicals. In three facilities, dust masks were occasionally used while working with green beans. In six facilities, hearing protection was available for voluntary use.

### Work Area and Workforce Description

The work areas and workforce were divided into three main groups of activities and site: production (e.g., administrative production, roaster, production, production support, quality control, grinder, flavoring, and packaging), non-production (e.g., administrative non-production), and café (e.g., barista and other café) to segregate the exposure groups into general areas of roasted coffee production, administration and support activities, or cafés, respectively. Work areas within these main groups were consistent regardless of whether the facility flavored coffee during the survey (Supplementary Table 3). Consistent work areas among facilities were roasting, grinding, packaging, shipping, and storage, with differences among facilities arising from individual facility layouts and level of segregation of processes. Some additional work areas were only present in flavoring facilities (e.g., flavoring). Workers duties necessitated movement throughout the facility to perform tasks in different areas, or the facility was small and open, meaning workers had the opportunity to be exposed to multiple emissions sources during their shift. Many facilities were small to medium size based on total number of production and non-production workers (range: 4–150) and had facility designs with occasional segregation of production/non-production spaces and shared general exhaust ventilation. No local exhaust ventilation was intentionally used for controlling exposures, but the roasting machines had exhausts that were sent outside the building; most facilities had downdraft cooling bins for roasted beans that also incidentally contributed to exposure mitigation. Administrative areas were sometimes within the main production area especially for smaller facilities with little to no separation of workspaces. Industrial hygienists, who were present during the sampling, assigned the workforce to job exposure groups (administrative non-production, administrative production, barista, flavoring, grinder, other café, packaging, production, production support, quality control, roaster) based on job title, job description, and whether they spent a majority of their time in the production area of the facility (Supplementary Table 4). Job exposure groups were assigned to group workers with similar job duties and potential for exposure. Workers who could not be assigned to a single job group because they performed multiple jobs were assigned to the generic production job exposure group.

### Sampling Approach

Monitoring at each facility was initiated by an HHE request. Outdoor full-shift area samples for alpha-diketones were collected to ensure ambient air was not contributing to workplace air. At each facility, workers were asked to voluntarily participate in the exposure assessment. Some workers were monitored multiple times over the course of the sampling campaign, which lasted 2 to 4 days depending on the facility. Repeat samples were collected for full-shift (over multiple days), task (on the same day and over multiple days), and instantaneous samples (on the same day and over multiple days) whenever possible. Personal sampling of the worker's breathing zone consisted of full-shift, task-based, and instantaneous samples for diacetyl and 2,3-pentanedione to identify tasks and processes that contributed to exposures. Area samples were located throughout the facility to assess chemical air concentrations in work areas using full-shift samples and from emission sources using instantaneous samples. Full-shift area samples were collected using area baskets placed at breathing height. Short-duration task samples were collected over several minutes and instantaneous samples over seconds to identify peak exposures and sources of diacetyl and 2,3-pentanedione. We collected one field blank per 17 samples and we extracted one media blank per 20 samples.

### Full-Shift and Task-Based Air Sampling and Analysis

We collected 415 personal and 760 area full-shift air samples for diacetyl (CAS No. 431-03-8), 2,3-pentanedione (CAS No. 600-14-6), 2,3-hexanedione (CAS No. 3848-24-6), and acetoin (CAS No. 513-86-0) on silica gel sorbent tubes (SKC, Inc., Eighty Four, PA). Samples were collected and analyzed according to the modified OSHA Sampling and Analytical Methods 1013/1016 (28–30). Two glass silica gel sorbent tubes were connected with tubing and inserted into a protective, light-blocking cover and sampled at a flow rate of 50 mL/min. For full-shift sampling, we collected two consecutive 3 h samples and calculated the time-weighted average (TWA) concentration, assuming the total 6 h monitoring results reflected a full work shift (8 h) TWA exposure. We refer to these samples as “full-shift samples” throughout this paper. We also collected 606 personal, short-term, task-based samples in the same manner over a median of 15 min (range: 2–86 min), at a flow rate of 200 mL/min as detailed in OSHA Methods 1013/1016 (28, 29).

Sample analyses were performed in the NIOSH Respiratory Health Division's Organics Laboratory. The samples were extracted for 1 h in 95% ethanol:5% water containing 3-pentanone as an internal standard. Samples were analyzed using an Agilent (Santa Clara, CA) 7890/7001 or 7890/5977 gas chromatograph/mass spectrometer (GC/MS) system operated in selected ion monitoring mode for increased sensitivity compared with the traditional flame ionization detector used in OSHA Methods 1013 and 1016 (30).

The median limits of detection (LODs) and limits of quantitation (LOQs) were 0.3 ppb and 1.0 ppb for diacetyl, 0.3 and 1.0 ppb for 2,3-pentanedione, 0.5 and 1.7 ppb for 2,3-hexanedione, and 1.5 and 5.0 ppb for acetoin for a typical full-shift air sample. The LODs and LOQs for task samples were typically three times higher than full-shift sample LOD and LOQ values because the air volumes collected during task samples were lower. Measurements below the LOD represent values that cannot reliably be distinguished from background noise, while measurements between the LOD and LOQ have a false positive probability of ~1% but the values have more uncertainty than measurements above the LOQ (31).

### Instantaneous Air Sampling and Analysis

We collected 35 pairs of pre- and post-shift instantaneous background air samples in the main production space to identify trends in background VOC levels over the workday, 296 instantaneous activity-based, and 312 instantaneous source air samples using evacuated canisters for diacetyl, 2,3-pentanedione, 2,3-hexanedione, and other VOCs in our standard calibration mixture: acetaldehyde, acetonitrile, ethanol, isopropyl alcohol, acetone, n-hexane, chloroform, methylene chloride, methyl methacrylate, benzene, ethylbenzene, toluene, styrene, *m, p*-xylene, *o*-xylene, α-pinene, and *d*-limonene. The sampler consisted of a 450-mL evacuated canister (Entech Instruments, Inc., Simi Valley, CA) equipped with an instantaneous fitting designed for a short sampling duration (<30 s). For activity-based air samples, a NIOSH investigator placed the inlet of the flow controller by the worker's breathing zone while they were performing a work activity. For source air samples, we placed the inlet of the flow controller directly at the source of interest.

Canister air samples were analyzed using a pre-concentrator/GC/MS system, with the following modifications: the pre-concentrator was a Model 7200 (Entech Instruments, Inc.); the GC/MS was an Agilent 7890/5977; and six additional compounds, diacetyl, 2,3-pentanedione, 2,3-hexanedione, acetaldehyde, acetonitrile, and styrene, were included in the calibration (32, 33). The median LODs for all the VOCs quantified are reported in Supplementary Table 5, and were 0.6 ppb for diacetyl, 0.8 ppb for 2,3-pentanedione, and 1.4 ppb for 2,3-hexanedione, based on a 1.5-times dilution factor, which is typical for instantaneous samples. However, individual LOD concentrations varied because they depended on the sample volume inside each canister.

### Data Analysis

Statistical analyses were performed using R 3.5.2 (R Foundation for Statistical Computing), JMP 12.0 and SAS 9.4 (SAS Institute, Inc., Cary, NC). Data were log-transformed before statistical analysis. The minimum, maximum, mean and coefficient of variation of the difference between pre- and post-shift diacetyl and 2,3-pentanedione instantaneous concentrations (post minus pre) were calculated. The relationship between log-transformed diacetyl and 2,3-pentanedione concentrations in full-shift personal and area samples was evaluated using linear regression modeling. Summary statistics including geometric means (GM), geometric standard deviations (GSD), and 95th percentile estimates (P95) were calculated using a Bayesian approach that simultaneously accounts for censored data (34). Bayesian computations were conducted using RJAGS/JAGS program in R (35). This approach fits a repeated measures analysis of variance (ANOVA) which accounts for repeated measurements collected on workers when at least five workers are present and at least two workers have repeated measurements. To keep the within- and between-subject GSDs in a reasonable range (1.01–50) because of small sample size, the within- and between-subject standard deviations (on the natural log scale) had uniform priors ranging from log(1.01) to log(50). The prior on the mean was left vague to allow the data to drive the inference, i.e., normal distribution prior mean 0 and variance 1,000,000. When analyzing area measurements including canister measurements, a one-way analysis of variance model was fit for each individual group of interest without the individual level random effect. This model contained the same normal prior on the mean component but had a vague/weakly-informative inverse-gamma prior on the variance component with shape = 0.1 and rate = 0.1, to allow for higher GSDs that are possible in canister measurements. Convergence was immediate for both models. To ensure convergence, we used 20,000 iterations (20,000 posterior samples of each quantity) after 5,000 iterations of burn-in were removed. While the Bayesian method provides distributions of parameters of interest (GM, GSD, P95), we only report the median values in the tables and text for simplicity; additional data on credible intervals for these parameters can be obtained upon request. For exposure groups with fewer than five observed measurements (non-censored), summary statistics were not calculated and only the maximum observation is reported in tables under P95 column heading. The AIHA exposure assessment strategy of comparing group-level P95 exposure estimates to the RELs was used as an approach to identify groups with potential for exposures exceeding the REL thus identifying particular job groups within coffee roasting facilities and cafés that are out of compliance with the REL (36). The P95 applies to all workers within a defined group and represents the exposure distribution of the group as it incorporates the mean and variance of the log-transformed exposures. The fraction of measurements above the NIOSH RELs were also calculated where appropriate. Given similarity in toxicological endpoint of diacetyl and 2,3-pentanedione exposures, the ACGIH^®^ additive mixture formula was used to calculate a mixed exposure index as the summation of the quotients of diacetyl and 2,3-pentanedione exposures to their respective REL (Concentration_diacetyl_/REL_diacetyl_ + Concentration_2, 3−pentanedione_/REL_2,3−pentanedione_). When this index exceeds 1.0, the mixture index has been exceeded (37); we use a generic term mixture index here as NIOSH has not specified an approach to compare exposure mixtures to RELs. A heatmap was generated to display the distribution of the mean concentration (log-transformed ppb) of eight VOCs collected by instantaneous activity or source samples during different production activities.

## Results

### 1013/1016 Field and Media Blanks

Analyte mass detected on the field blanks was low for most tubes (diacetyl < LOD to 0.092 μg/mL; 2,3-pentanedione < LOD to 0.056 μg/mL; 2,3-hexanedione < LOD for all; acetoin < LOD for all but one sample that measured at 5.6 μg/mL and was likely contaminated in the field). Analyte mass detected on media blanks was low (diacetyl < LOD for all; 2,3-pentanedione < LOD for all; 2,3-hexanedione < LOD to 0.16 μg/mL; acetoin < LOD to 0.045 μg/mL). No field blank or media blank correction was performed.

### 1013/1016 Outdoor Full-Shift Concentrations

Outdoor full-shift samples had low concentrations of diacetyl (<0.3 ppb for all non-flavoring facilities, with 100% below LOD; <0.3 to 14.1 ppb for flavoring facilities, with 46% below LOD; <0.3 to 0.42 ppb for cafés, with 83% below LOD) and 2,3-pentanedione (<0.3 ppb for all non-flavoring facilities, with 100% below LOD; <0.3 to 0.5 ppb for flavoring facilities and cafés with 60% below LOD for flavoring and 83% below LOD for cafés). Outdoor samples were also mostly non-detectable for 2,3-hexanedione (100% below LOD for flavoring and non-flavoring facilities; 93% below LOD for cafés) and acetoin (100% below LOD for flavoring and non-flavoring facilities; 86% below LOD for cafés).

### Instantaneous Background Area Concentrations

Background air concentrations of diacetyl and 2,3-pentanedione increased between pre- and post-shift canister samples in cafés and production facilities because of activities during the work-shift (Table 2). In cafés, the mean increase was 2.3 ppb for diacetyl and 2.4 ppb for 2,3-pentanedione. In production areas of non-flavoring facilities, the mean increase was 8.4 ppb for diacetyl and 4.5 ppb for 2,3-pentanedione. In production areas of flavoring facilities, the mean increase was 2.9 ppb for diacetyl and 8.3 ppb for 2,3-pentanedione. All mean differences in cafés and in production areas were significantly greater than zero (*p* < 0.01).

**Table 2:** Average difference between pre- and post-shift background diacetyl and 2,3-pentanedione concentrations using instantaneous evacuated canisters (NMAM 3900) by production area.

**Production area**	**Analyte**	**N**	**Average difference (ppb)**	**CV**	**Minimum difference (ppb)**	**Maximum difference (ppb)**
**NON-FLAVOR**						
Café	Diacetyl	4	2.4	86.5	0.8	5.5
Café	2,3-Pentanedione	4	2.3	88.4	0.6	5.2
Production	Diacetyl	11	8.4	106.3	0.0	28.4
Production	2,3-Pentanedione	11	4.5	105.3	0.0	16.6
**FLAVOR**						
Non-production	Diacetyl	1	–	–	2.3	2.3
Non-production	2,3-Pentanedione	1	–	–	3.9	3.9
Production	Diacetyl	19	2.9	337.0	−16.4	22.0
Production	2,3-Pentanedione	19	8.3	111.8	−3.8	24.1

*N, number of samples; ppb, parts per billion; CV, coefficient of variation; “–,” No mean or CV for one sample*.

### Full-Shift Personal Exposures

**Figure 1:**
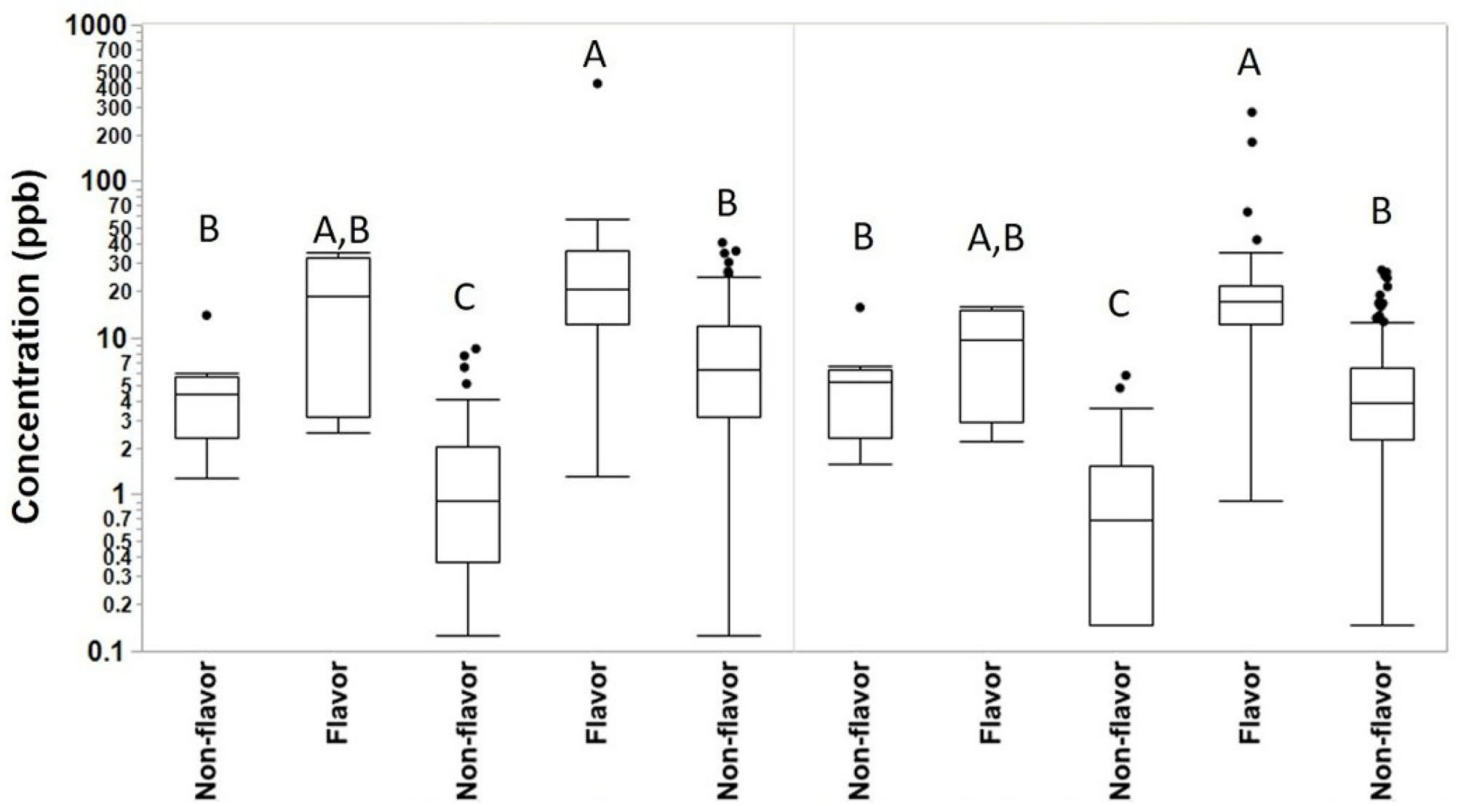
Full-shift TWA personal exposures to diacetyl and 2,3-pentanedione among café, production and non-production workers in flavoring and non-flavoring facilities in samples analyzed using modified OSHA Method 1013/1016. From left to right, number of samples *n* = 18 for café, *n* = 6 for non-production flavoring, *n* = 41 for non-production non-flavoring, *n* = 91 for production flavoring, and *n* = 259 for production non-flavoring. By compound, connecting letters indicate groups not statistically different.

We collected 415 personal full-shift exposures to diacetyl, 2,3-pentanedione, 2,3-hexanedione and acetoin from 227 workers. These exposures were typically higher among production workers than non-production workers and higher among workers in flavored coffee facilities compared to non-flavored coffee facilities (Table 3, Figure 1). Exposures to diacetyl were lowest in non-production workers of facilities that did not flavor coffee (GM 0.9 ppb; P95 6.0 ppb). Exposures to diacetyl were highest in facilities that flavored coffee regardless of production or non-production status of the worker. For example, exposures to diacetyl were not statistically different (Figure 1) between production workers (GM 21 ppb; P95 79 ppb) and non-production workers (GM 11 ppb; P95 92 ppb) in facilities that flavored coffee (Table 3). Exposures to 2,3-pentanedione were also lowest in non-production workers of facilities that did not flavor coffee (GM 0.7 ppb; P95 4.4 ppb) and highest in production workers (GM 15 ppb; P95 52 ppb) and non-production workers (GM 7.1 ppb; P95 33 ppb) of facilities that flavored coffee. There was no statistical difference observed between production and non-production workers in flavoring facilities (Figure 1). Exposures were above the REL for diacetyl in 95% of the samples and for 2,3-pentanedione in 77% of the samples collected among production workers of flavoring facilities. Exposures to acetoin were mostly non-detectable (≥88% below LOD) in non-flavored coffee facilities, but elevated (GM 27 ppb; P95 413 ppb) in production areas of flavor facilities. Exposures to 2,3-hexanedione were mostly below the LOD (flavoring production 77% < LOD; non-flavoring production 92% < LOD) (Table 3).

**Table 3:** Personal TWA exposures to diacetyl, 2,3-pentanedione, acetoin, and 2,3-hexanedione using modified OSHA Methods 1013/1016 by production area.

**Production area**	**Analyte**	**N**	**k**	**GM (ppb)**	**GSD**	P95 or max^*^ (ppb)	**%BDL**	**% Above REL**
**NON-FLAVOR**								
Café	Diacetyl	18	17	3.9	1.8	10	0	44
Non-production	Diacetyl	41	26	0.9	3.1	6	22	9.8
Production	Diacetyl	259	130	5.6	2.5	25	7	62
Café	2,3-Pentanedione	18	17	4.4	1.8	12	0	5.6
Non-production	2,3-Pentanedione	41	26	0.7	3	4.4	27	0
Production	2,3-Pentanedione	259	130	3.6	2.3	14	7	10.8
Café	Acetoin	18	17	–	–	2.7^*^	89	–
Non-production	Acetoin	41	26	0	15	3.1	88	–
Production	Acetoin	259	130	1	2.1	3.5	63	–
Café	2,3-Hexanedione	18	17	–	–	–	100	–
Non-production	2,3-Hexanedione	41	26	–	–	0.5^*^	95	–
Production	2,3-Hexanedione	259	130	0.1	3.5	0.5	92	–
**FLAVOR**								
Non-production	Diacetyl	6	4	11	3.7	92	0	67
Production	Diacetyl	91	52	21	2.2	79	0	95
Non-production	2,3-Pentanedione	6	4	7.1	2.5	33	0	50
Production	2,3-Pentanedione	91	52	15	2.1	52	0	77
Non-production	Acetoin	6	4	12	13	763	17	–
Production	Acetoin	91	52	27	5.3	413	6	–
Non-production	2,3-Hexanedione	6	4	–	–	–	100	–
Production	2,3-Hexanedione	91	52	0.1	4.6	1.5	77	–

TWA, time-weighted average; N, number of samples; k, number of workers; GM, geometric mean; ppb, parts per billion; GSD, geometric standard deviation; P95, 95th percentile; %BDL, percent samples below the limit of detection; max^*^, maximum presented when <5 measurements were above the detection limit; %Above REL, percentage above NIOSH recommended exposure limit; “–,” not enough samples above the detection limit to obtain an estimate or no REL.

Flavoring facilities had the highest percentage of full-shift personal exposures exceeding the mixture index (Table 4). The flavor/non-production group exceeded the mixture index in 83% of samples compared to 12% in the non-flavor/non-production group. The difference in flavoring status was not as prominent when comparing the flavor/production group (96%) to non-flavor/production (73%). Full-shift exposures from cafés exceeded the mixture index in 67% of samples.

**Table 4:** Percent of full-shift TWA personal exposures exceeding the mixture index for diacetyl and 2,3-pentanedione using modified OSHA Methods 1013/1016 by production area.

**Production area**	***N***	**k**	**N (%) exceeding mixture index of 1.00**	**Median (min, max) mixture indices that exceeded 1.00**
**NON-FLAVOR**				
Café	18	17	12 (67)	1.73 (1.04–4.47)
Non-production	41	26	5 (12)	1.69 (1.08–2.32)
Production	259	130	187 (72)	2.45 (1.00–11.0)
**FLAVOR**				
Non-production	6	4	5 (83)	7.65 (1.00–8.69)
Production	91	52	87 (96)	6.37 (1.34–114.0)

*N, number of samples; k, number of workers; min, minimum; max, maximum*.

Personal full-shift exposures were higher among job groups that packaged, ground, or flavored roasted coffee such as grinder operator, packaging worker, production worker, and quality control worker (Table 5) compared with administrative workers and roaster operators. For flavored coffee facilities, personal full-shift exposures were highest among flavoring workers (GM 34, P95 284 ppb diacetyl; GM 38, P95 348 ppb 2,3-pentanedione) followed by packaging worker (GM 27, P95 54 ppb diacetyl; GM 19, P95 32 ppb 2,3-pentanedione) and grinder operator (GM 26, P95 102 ppb diacetyl; GM 22, P95 76 ppb 2,3-pentanedione). For non-flavor coffee facilities, personal full-shift exposures were generally lower than in flavored coffee facilities with the highest exposures observed among packaging workers (GM 8.0, P95 26 ppb diacetyl; GM 4.4, P95 12 ppb 2,3-pentanedione), followed by quality control worker (GM 6.4, P95 18 ppb diacetyl; GM 3.8, P95 13 ppb 2,3-pentanedione) and production workers (GM 6.3, P95 24 ppb diacetyl; GM 4.6, P95 18 ppb 2,3-pentanedione). Baristas in cafés had average full-shift exposures below the RELs (GM 4.1 ppb diacetyl; GM 4.6 ppb 2,3-pentanedione) but P95 above the REL (11.0 ppb diacetyl; 13.0 ppb 2,3-pentanedione) and 64% above the mixture index. For non-flavor, administrative non-production workers had the lowest exposures (GM 0.9, P95 4.4 ppb diacetyl; GM 0.6, P95 3.3 ppb 2,3-pentanedione) and the lowest percentage above the mixture index (7.9%). Exposures to acetoin and 2,3-hexanedione by job group are summarized in Supplementary Table 6; acetoin exposures were highest among flavoring workers (GM 163 ppb; P95 5,622 ppb). Exposures to 2,3-hexanedione were mostly non-detectable.

**Table 5:** Personal TWA exposures to diacetyl and 2,3-pentanedione using modified OSHA Methods 1013/1016 by job group.

			**Diacetyl**				**2,3-Pentanedione**				
**Job Group**	***N***	**k**	**GM (ppb)**	**GSD**	**P95 (ppb)**	**%BDL**	**GM (ppb)**	**GSD**	**P95 (ppb)**	**%BDL**	**% Above Mixture Index**
**NON-FLAVOR**											
Administrative non-production worker	38	23	0.9	2.7	4.4	21	0.6	2.8	3.3	29	7.9
Administrative production worker	53	25	2.9	3.3	21	19	2.0	3.0	12	17	47
Barista	14	13	4.1	1.9	11	0	4.6	1.9	13	0	64
Grinder operator	3	3	–	–	11^*^	0	–	–	6.2^*^	0	67
Other café worker	7	7	4.0	1.9	11	0	3.8	1.6	8.6	0	71
Packaging worker	80	41	8.0	2.0	26	5	4.4	1.9	12	5	84
Production worker	36	24	6.3	2.3	24	0	4.6	2.3	18	0	81
Production support worker	9	4	5.2	3.2	35	11	3.2	1.7	7.5	0	89
Quality control worker	15	5	6.4	1.9	18	0	3.8	2.1	13	0	87
Roaster operator	63	34	5.1	2.6	24	6.3	3.3	2.4	14	7.9	68
**FLAVOR**											
Administrative non-production worker	7	5	5.1	12	279	14	4.4	4.9	59	0	71
Administrative production worker	6	3	12	2.2	42	0	10	2.0	33	0	100
Flavoring worker	7	4	34	3.7	284	0	38	3.9	348	0	100
Grinder operator	5	2	26	2.3	102	0	22	2.1	76	0	100
Packaging worker	44	27	27	1.5	54	0	19	1.4	32	0	100
Production worker	5	3	4.3	2.4	18	0	4.0	2.7	21	0	60
Production support worker	3	2	–	–	36^*^	0	–	–	17^*^	0	100
Quality control worker	4	4	–	–	37^*^	0	–	–	18^*^	0	100
Roaster operator	17	7	15	2.8	81	0	9.4	2.4	39	0	88

TWA, time-weighted average; N, number of samples; k, number of workers; GM, geometric mean; ppb, parts per billion; GSD, geometric standard deviation; P95, 95th percentile; %BDL, percent samples below the limit of detection; % Above Mixture Index, percentage above mixture index; max^*^, maximum presented when <5 measurements were above the detection limit; “–,” not enough samples above the detection limit to obtain an estimate.

### Full-Shift Area Concentrations

We collected 760 full-shift area air concentrations for alpha-diketones. Area air concentrations of diacetyl and 2,3-pentanedione were higher in the production and non-production areas of the flavoring facilities compared to non-flavoring (Table 6). The non-production area measurements of non-flavoring facilities were the lowest, followed by cafés and production areas.

**Table 6:** Area TWA concentrations of diacetyl, 2,3-pentanedione, acetoin, and 2,3-hexanedione using modified OSHA Methods 1013/1016 by production area.

**Production area**	**Analyte**	***N***	**GM (ppb)**	**GSD**	P95 or max^*^ (ppb)	**%BDL**
**NON-FLAVOR**						
Café	Diacetyl	52	2.4	3.1	15	9.6
Café	2,3-Pentanedione	52	2.7	2.6	13	1.9
Café	Acetoin	52	–	–	5.0*	92
Café	2,3-Hexanedione	52	–	–	0.9*	98
Non-production	Diacetyl	72	1.2	3.0	7.1	22
Non-production	2,3-Pentanedione	72	0.8	2.9	4.7	29
Non-production	Acetoin	72	0.2	4.1	1.8	92
Non-production	2,3-Hexanedione	72	–	–	–	100
Production	Diacetyl	380	4.9	3.5	38	8.7
Production	2,3-Pentanedione	380	3.1	3.2	22	12
Production	Acetoin	380	1.2	2.3	4.7	55
Production	2,3-Hexanedione	380	0.04	4.9	0.6	92
**FLAVOR**						
Non-production	Diacetyl	32	8.3	3.3	59	0
Non-production	2,3-Pentanedione	32	3.2	7.2	81	19
Non-production	Acetoin	32	5.0	8.9	182	28
Non-production	2,3-Hexanedione	32	–	–	0.5^*^	94
Production	Diacetyl	224	21	2.5	94	0
Production	2,3-Pentanedione	224	16	2.4	70	0
Production	Acetoin	224	29	6.5	628	4.9
Production	2,3-Hexanedione	224	0.1	6.4	1.1	87

TWA, time-weighted average; N, number of samples; GM, geometric mean; ppb, parts per billion; GSD, geometric standard deviation; P95, 95th percentile; %BDL, percent samples below the limit of detection; max^*^, maximum presented when <5 measurements were above the detection limit; “–,” not enough samples above the detection limit to obtain an estimate.

Proximity to a source such as roasted coffee or flavoring influenced air concentrations of diacetyl and 2,3-pentanedione (Table 7). Bakery/Cafés had low (although not the lowest) average area air concentrations of diacetyl (GM 2.5 ppb; P95 15 ppb) and 2,3-pentanedione (GM 2.8 ppb; P95 13 ppb). For production areas of non-flavoring facilities, grinding area had the highest diacetyl GM of 12 ppb but was variable (GSD 3.2) compared with packaging area with a diacetyl GM of 8.6 ppb (GSD 2.1). Flavoring facilities had higher area concentrations of diacetyl and 2,3-pentanedione in production areas than non-flavoring facilities (diacetyl GM 17 ppb vs. 3.0 ppb; 2,3-pentanedione GM 14 ppb vs. 2.0 ppb). Flavoring area had the highest area GMs of 33 ppb for diacetyl (P95 235 ppb) and 49 ppb for 2,3-pentanedione (P95 456 ppb). Acetoin area concentrations were generally higher in production areas of flavoring facilities compared to non-flavoring facilities (GM 29 ppb vs. GM 1.2 ppb; Table 6) and highest in flavoring areas of flavoring facilities (GM 304 ppb; P95 9,440 ppb; Supplementary Table 7). Area concentrations of 2,3-hexanedione were mostly observed at low concentrations in flavoring areas and in grinding areas within both flavoring and non-flavoring facilities (Supplementary Table 7).

**Table 7:** Area TWA concentrations of diacetyl and 2,3-pentanedione using modified OSHA Methods 1013/1016.

		**Diacetyl**				**2,3-Pentanedione**			
**Area**	**N**	**GM (ppb)**	**GSD**	**P95 (ppb)**	**%BDL**	**GM (ppb)**	**GSD**	**P95 (ppb)**	**%BDL**
**NON-FLAVOR**									
Administration area	63	1.0	2.8	5.7	19	0.7	2.9	3.8	29
Bakery/café	54	2.5	3.1	15	9.3	2.8	2.6	13	1.9
Breakroom	9	2.2	2.1	7.2	33	1.6	1.9	4.8	33
Green bean storage area	7	0.9	2.9	4.9	14	–	–	2.1^*^	43
Grinding area	40	12	3.2	81	7.5	7.5	3.0	44	7.5
Packaging area	103	8.6	2.1	29	1	5.0	2.1	17	1
Production area	102	3.0	3.3	22	7.8	2.1	2.8	11	8.8
Production storage area	25	3.6	4.2	38	20	2.0	5.5	33	28
Quality control area	20	2.1	3.1	14	15	1.8	2.4	7.8	15
Roasting area	72	5.2	3.4	39	18	3.3	3.3	23	21
Shipping area	9	2.3	4.1	23	0	0.8	8.4	26	33
**FLAVOR**									
Administration area	21	8.6	3.6	72	0	6.7	2.9	39	0
Breakroom	7	13	3.1	84	0	8.9	2.5	40	0
Flavoring area	19	33	3.3	235	0	49	3.9	456	0
Green bean storage area	12	14	2.4	60	0	10	2.0	32	0
Grinding area	26	25	2.5	113	0	18	2.3	68	0
Packaging area	87	24	1.8	66	0	19	1.6	43	0
Production area	12	17	2.0	51	0	14	1.7	32	0
Production storage area	35	16	3.3	108	0	5.5	8.0	168	17
Quality control area	7	15	2.4	62	0	10	1.7	26	0
Roasting area	30	13	3.2	90	0	9.2	2.8	49	0

TWA, time-weighted average; GM, geometric mean; ppb, parts per billion; GSD, geometric standard deviation; P95, 95th percentile; %BDL, percent samples below the limit of detection; max^*^, maximum presented when <5 measurements were above the detection limit; “–,” not enough samples above the detection limit to obtain an estimate.

### Comparison of Diacetyl and 2,3-Pentanedione

**Figure 2:**
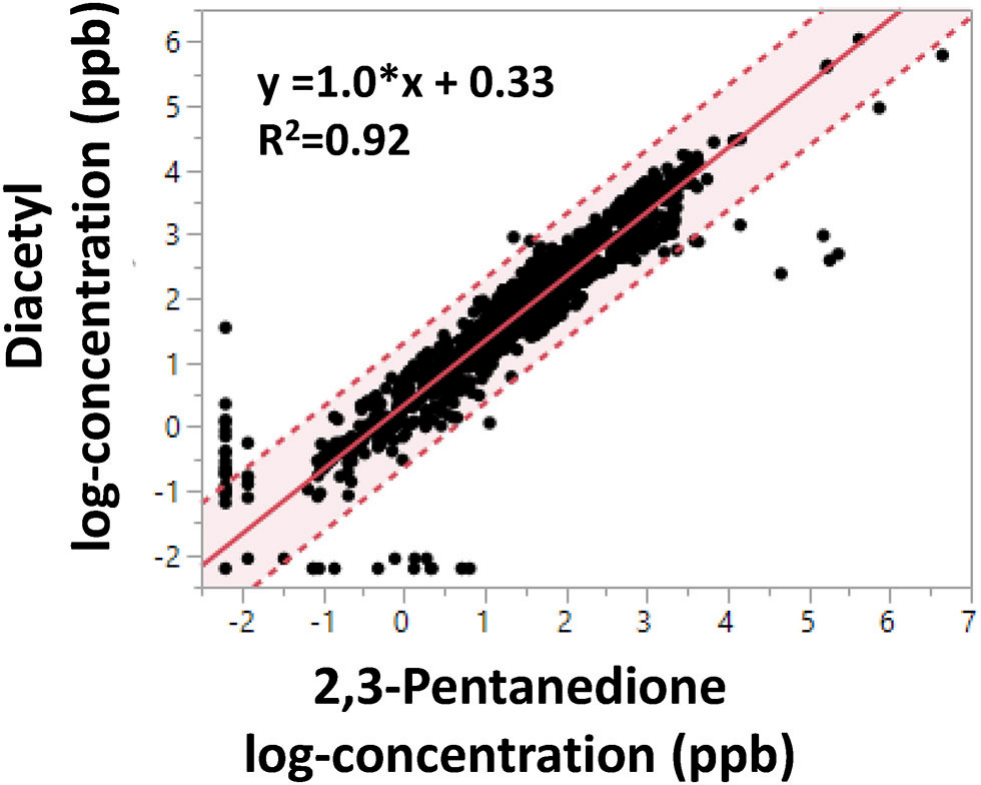
Linear regression of OSHA Methods 1013/1016 diacetyl and 2,3-pentanedione air concentrations (log-concentration in ppb). Shaded area indicates 95% confidence interval. Dotted lines represent 95% confidence limits.

Linear regression of log-transformed diacetyl and 2,3-pentanedione air concentrations (*n* = 1,175, personal and area samples) revealed a positive association with a slope of 1.0, a positive y-intercept of 0.33 and a coefficient of determination of 0.92 (Figure 2). The regression indicates diacetyl and 2,3-pentanedione air concentrations track well together. Similar trends and estimates were obtained when the regression model was stratified by facility, flavoring use, or personal vs. area sample type (data not shown). Similar trends were expected among facilities and between sample types because both diacetyl and 2,3-pentanedione are naturally produced and emitted during roasting, grinding and packaging coffee beans. However, differences may arise between measurements of flavored and non-flavored coffee depending on the addition of different flavoring products.

### Personal Task Exposures

We collected 606 personal task-based exposure measurements from 134 workers. Exposure to alpha-diketones during short-duration tasks were highest when moving, grinding or flavoring roasted coffee (Table 8, Supplementary Table 8). Grinding coffee beans had the highest personal task exposure for both non-flavored coffee (GM 26, P95 181 ppb diacetyl; GM 20, P95 109 ppb 2,3-pentanedione) and flavored coffee (GM 30, P95 166 ppb diacetyl; GM 31, P95 205 ppb 2,3-pentanedione) facilities (Table 8). Moving roasted beans or ground coffee had the second highest task exposure in non-flavored coffee facilities (GM 20, P95 142 ppb diacetyl; GM 11, P95 80 ppb 2,3-pentanedione). Flavoring coffee had the highest P95 exposures to alpha-diketones (GM 5.4, P95 1,102 ppb diacetyl; GM 45, P95 3,816 ppb 2,3-pentanedione). Packaging coffee task exposures in flavored coffee facilities was higher than in non-flavored coffee facilities for diacetyl (diacetyl GM 25, P95 71 ppb vs. GM 8.6, P95 46 ppb) and 2,3-pentanedione (GM 15, P95 59 ppb vs. GM 5.3, P95 26 ppb). Exposures to acetoin were higher for tasks in flavored coffee facilities than in non-flavored coffee facilities, which had 60–100% of measurements below the LOD with the exception of packaging rework tasks (25% < LOD) (Supplementary Table 8). High acetoin peak exposures, reflected by the P95 estimates (range: GM 2.1–29 ppb, P95 11–8,969 ppb), occurred for multiple tasks in flavoring. Exposures to 2,3-hexanedione for tasks were mostly low with 73–100% of measurements below the LOD for the tasks in flavoring and non-flavoring facilities, except for the task of packaging rework (25% < LOD).

**Table 8:** Personal task exposures to diacetyl and 2,3-pentanedione using modified OSHA Methods 1013/1016.

				**Diacetyl**				**2,3-Pentanedione**			
**Task**	**Sampling time (min-max)**	***N***	**k**	**GM (ppb)**	**GSD**	**P95 (ppb)**	**%BDL**	**GM (ppb)**	**GSD**	**P95 (ppb)**	**%BDL**
**NON-FLAVOR**											
Miscellaneous café tasks	5–16	10	6	2.2	4.5	25	30	3.5	2.5	16	10
Cleaning machines	7–20	9	6	3.4	7.4	89	22	1.7	9.5	59	33
Grinding coffee beans	2–18	58	25	26	3.2	181	5.2	20	2.8	109	1.7
Maintenance of machines	13–15	5	1	–	–	15^*^	20	–	–	7.8^*^	20
Miscellaneous production	3–29	9	5	4.0	6.1	78	11	2.2	5.4	34	22
Moving roasted beans or ground coffee	3–25	10	6	20	3.3	142	0	11	3.2	80	0
Packaging coffee	5–53	153	56	8.6	2.8	46	5.9	5.3	2.6	26	7.8
Quality control	4–18	40	9	2.2	6.9	45	33	4.8	2.2	18	2.5
Packaging rework	15–15	4	2	–	–	70^*^	0	–	–	39^*^	0
Roasting coffee beans	10–86	152	27	2.6	5.2	39	27	2.4	4.0	24	24
**FLAVOR**											
Cleaning machines	5–46	27	12	15	2.9	90	7.4	11	2.3	46	7.4
Flavoring coffee	6–18	15	5	5.4	30	1,102	27	45	15	3,816	6.7
Grinding coffee beans	7–32	19	9	30	2.8	166	0	31	3.1	205	0
Miscellaneous production	7–14	3	3	–	–	29^*^	33	–	–	15^*^	33
Moving roasted beans or ground coffee	4–15	3	3	–	–	43^*^	0	–	–	24^*^	0
Packaging coffee	3–55	46	18	25	1.9	71	2.2	15	2.3	59	8.7
Roasting coffee beans	7–30	43	8	11	3.7	94	16	7.7	3.3	55	21

N, number of samples; k, number of workers; GM, geometric mean; ppb, parts per billion; GSD, geometric standard deviation; P95, 95th percentile; %BDL, percent samples below the limit of detection; max^*^, maximum presented when <5 measurements were above the detection limit; “–,” not enough samples above the detection limit to obtain an estimate.

### VOC Canister Instantaneous Activity Exposures

**Figure 3:**
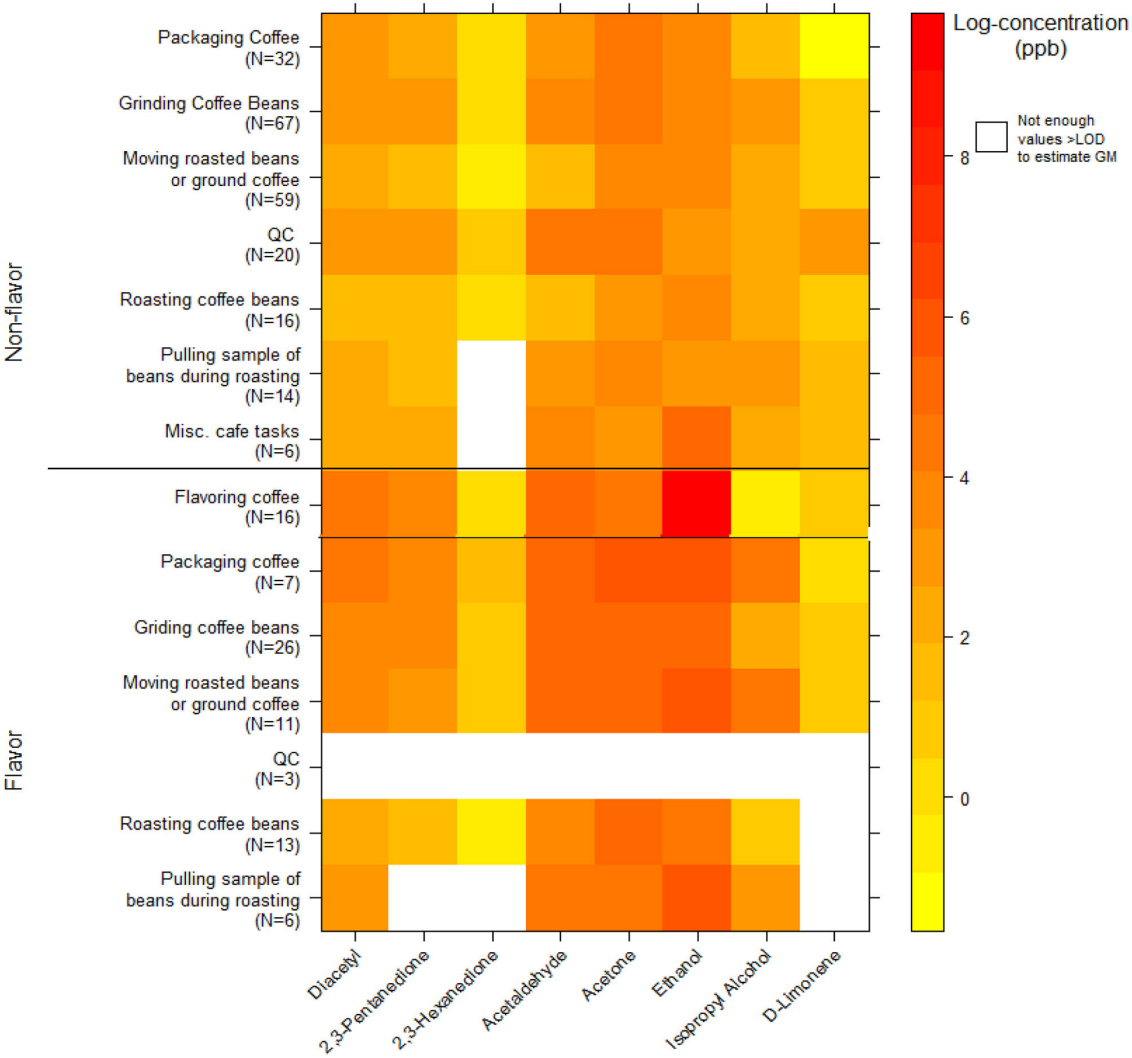
Heatmap of personal instantaneous activity exposures for select VOCs (log-concentration in ppb) using canisters (NMAM 3900).

We collected 296 instantaneous VOC canister activity air measurements. GMs for diacetyl and 2,3-pentanedione in production ranged from 3.7 ppb (2,3-pentanedione) for roasting coffee beans to 76 ppb (diacetyl) for packaging coffee in flavored coffee facilities (Table 9). The highest activity concentrations in flavored coffee facilities were flavoring coffee (GM 62, P95 5,311 ppb diacetyl) and in non-flavored coffee facilities were grinding coffee beans (GM 25, P95 314 ppb diacetyl). The distributions of all additional VOC mean activity exposures are displayed in a heat map (Figure 3). The highest measured exposure to additional VOCs was for ethanol during flavoring coffee, which was observed at a GM of 8,765 ppb (P95 263,320 ppb; GSD 8.0) (Supplementary Table 9). Acetaldehyde exposures while flavoring coffee varied widely (GM 156 ppb; GSD 8.1) and had a P95 concentration of 4,846 ppb, which is 5.4 times lower than the ACGIH TLV^®^ ceiling of 25 ppm. Acetaldehyde and acetone exposures were generally higher in flavored coffee facilities.

**Table 9:** Instantaneous activity exposures of diacetyl and 2,3-pentanedione using evacuated canisters (NMAM 3900).

	**Diacetyl**				**2,3-Pentanedione**				
**Activity**	**N**	**GM (ppb)**	**GSD**	**P95 (ppb)**	**%BDL**	**GM (ppb)**	**GSD**	**P95 (ppb)**	**%BDL**
**NON-FLAVOR**									
Grinding coffee beans	67	25	4.7	314	0	15	4.7	191	1.5
Miscellaneous café tasks	6	8.6	1.4	15	0	8.5	1.5	16	0
Moving roasted beans or ground coffee	59	9.3	6.7	212	3.4	5.8	7.6	164	17
Packaging coffee	32	18	2.7	90	0	13	3.0	78	0
Pulling sample of beans during roasting	14	8.4	3.0	51	0	5.2	3.0	32	7.1
Quality control	20	22	2.2	79	0	14	2.4	61	0
Roasting coffee beans	16	5.6	4.3	62	6.3	4.4	4.2	47	6.3
**FLAVOR**									
Flavoring coffee	16	62	15	5,311	6.3	54	7.9	1,594	0
Grinding coffee beans	26	42	6.6	933	7.7	46	3.1	299	0
Moving roasted beans or ground coffee	11	42	2.4	179	9.1	24	2.4	98	9.1
Packaging coffee	7	76	2.5	342	0	39	2.4	158	0
Pulling sample of beans during roasting	6	17	8.2	535	17	–	–	49^*^	33
Quality control	3	–	–	53^*^	0	–	–	30^*^	0
Roasting coffee beans	13	9.3	3.5	73.0	0	3.7	6.1	71	15

N, number of samples; GM, geometric mean; ppb, parts per billion; GSD, geometric standard deviation; P95, 95th percentile; %BDL, percent samples below the limit of detection; max^*^, maximum presented when <5 measurements were above the detection limit; “–,” not enough samples above the detection limit to obtain an estimate.

### VOC Canister Area Source Measurements

**Figure 4:**
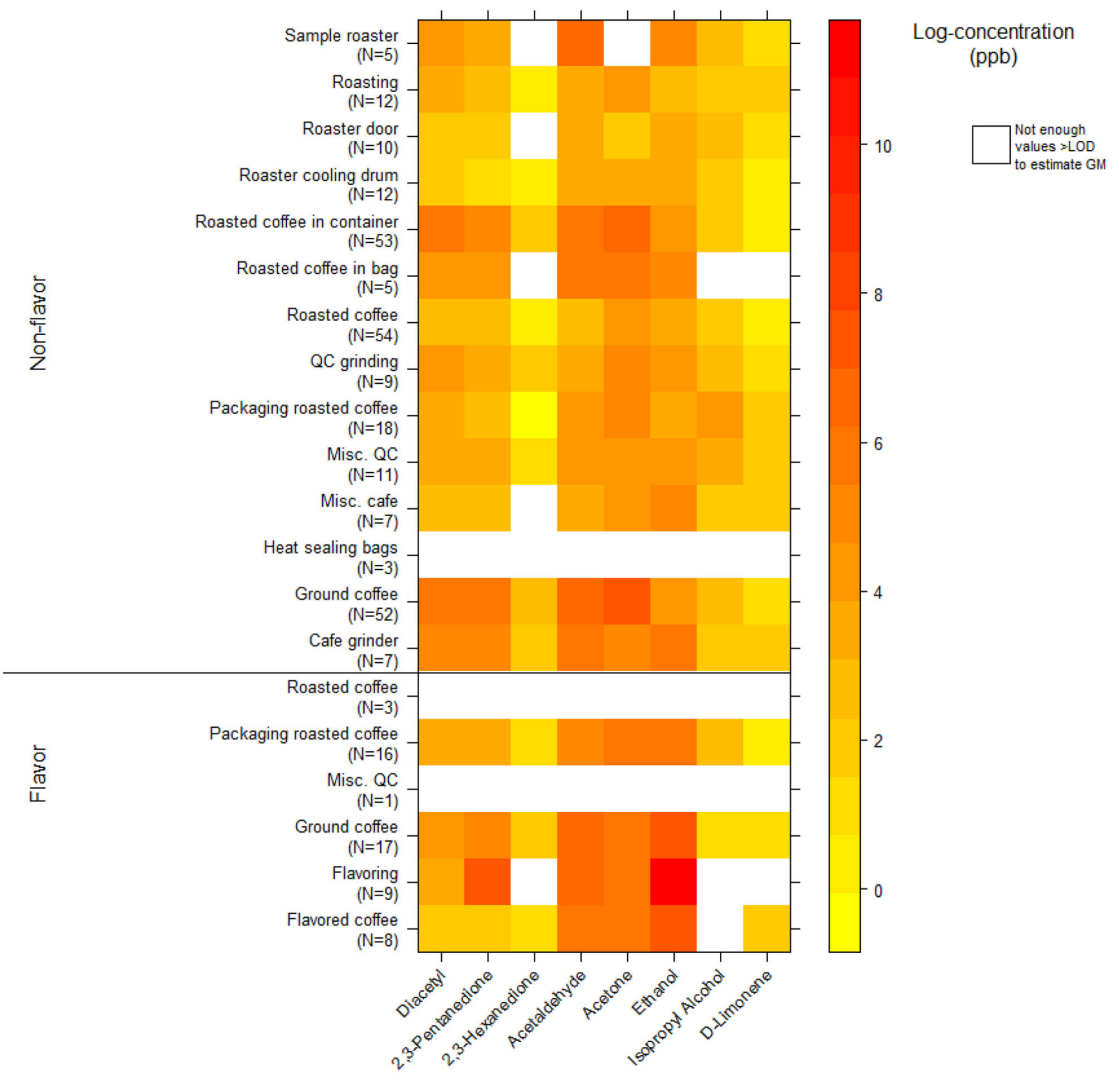
Heatmap of instantaneous area source concentrations for select VOCs (log-concentration in ppb) using canisters (NMAM 3900).

We collected 312 instantaneous source measurements. The highest emission sources of diacetyl and 2,3-pentanedione were roasted whole bean and ground coffee, grinding, and flavoring (Table 10). The two highest sources for diacetyl based on GM were ground coffee (GM 488, P95 21,788 ppb) and roasted coffee in a container (GM 225, P95 7,168 ppb), both in non-flavored coffee facilities. The two highest sources for 2,3-pentanedione based on GM were flavoring (GM 1,882, P95 185,446 ppb) and ground coffee (GM 251, P95 12,674 ppb, non-flavored coffee facility). The highest source for diacetyl and 2,3-pentanedione based on P95 was flavoring (P95 354,158 ppb diacetyl; P95 185,446 ppb 2,3-pentanedione). The distributions of instantaneous source means for alpha-diketones and other VOCs are displayed in a heat map (Figure 4). Acetaldehyde had highest emissions from ground coffee (GM 987, P95 42,631 ppb) and from roasted coffee in bag (GM 229, P95 52,991 ppb) in non-flavored facilities (Supplementary Table 10). Ethanol had highest emissions from flavoring (GM 54,154, P95 1.02 ×10^6^ ppb) in flavored coffee facilities (Supplementary Table 10). Acetone also had highest emissions from flavoring (GM 341, P95 301,886 ppb) and from ground coffee (GM 477, P95 38,147 ppb) in flavored coffee facilities (Supplementary Table 10).

**Table 10:** Area source concentrations of diacetyl and 2,3-pentanedione using evacuated canisters (NMAM 3900).

		**Diacetyl**				**2,3-Pentanedione**			
**Source**	**N**	**GM (ppb)**	**GSD**	**P95 (ppb)**	**%BDL**	**GM (ppb)**	**GSD**	**P95 (ppb)**	**%BDL**
**NON-FLAVOR**									
Café grinder	7	118	6.5	2,487	0	122	6.4	2,501	0
Ground coffee	52	488	10	21,788	0	251	11	12,674	0
Heat sealing bags	3	–	–	16*	0	–	–	8.8*	0
Miscellaneous quality control	11	27	4.9	366	0	22	5.6	368	0
Miscellaneous café	7	12	2.2	44	0	13	2.5	58	0
Packaging roasted coffee	18	28	4.2	292	0	14	3.9	129	0
Quality control grinding	9	50	6.0	928	0	42	5.7	720	0
Roasted coffee	54	19	4.7	245	0	10	4.6	125	1.9
Roasted coffee in bag	5	76	27	16,456	0	68	19	8,491	0
Roasted coffee in container	53	225	8.2	7,168	0	140	7.9	4,213	0
Roaster cooling drum	12	6.0	3.3	41	0	3.2	4.0	31	8.3
Roaster door	10	8.3	2.2	30	0	4.9	2.7	25	0
Roasting	12	21	6.0	411	8.3	11	4.4	123	8.3
Sample roaster	5	75	7.6	2,059	0	38	12	2,143	0
**FLAVOR**									
Flavored coffee	8	6.6	100	11,868	38	6.3	71	6,190	38
Flavoring	9	24	381	354,158	44	1,882	17	185,446	0
Ground coffee	17	59	36	20,945	24	143	9.7	6,038	5.9
Miscellaneous quality control	1	–	–	37*	0	–	–	20*	0
Packaging roasted coffee	16	45	4.4	497	13	47	3.6	378	6.3
Roasted coffee	3	–	–	7,386*	33	–	–	1,749*	0

N, number of samples; GM, geometric mean; ppb, parts per billion; GSD, geometric standard deviation; P95, 95th percentile; %BDL, percent samples below the limit of detection; –, not enough samples above the detection limit to obtain an estimate; max*, maximum presented when <5 measurements were above the detection limit; “–,” not enough samples above the detection limit to obtain an estimate.

## Discussion

To investigate the potential health effects of coffee emissions, we aggregated data from exposure assessments at flavored and non-flavored coffee production facilities and cafés associated with these facilities, through the NIOSH HHE program. The main sources of VOC exposures in coffee facilities and cafés were roasted coffee and flavorings. Roasted coffee contains a complex chemical mixture of over 850 compounds (38). Many of these compounds are VOCs including diacetyl, 2,3-pentanedione, and 2,3-hexanedione, and other chemicals such as CO and CO_2_, which are naturally produced when coffee beans are roasted (5–8, 11, 39–41). High CO source emissions were observed where coffee was stored and ground in a number of the facilities, and the results from one facility are discussed elsewhere (12). We observed varying concentrations of diacetyl relative to 2,3-pentanedione in the same air sample in non-flavoring facilities presumably because of differences in green beans and roasting practices among these facilities; coffee roast temperature and time affect aroma formation and VOC profiles (42). The ratio of diacetyl to 2,3-pentanedione concentrations from roasted coffee increases with increasing roasting temperature (400 to 430°F) (43). Volatile constituents are trapped inside the pore structure of the roasted coffee bean and rapidly released when coffee is ground because of the greater surface area for off-gassing (11). For flavored coffee facilities, we observed higher exposures to diacetyl and acetoin than 2,3-pentanedione compared to non-flavoring facilities presumably because of the composition of the bulk flavorings used at the time of sampling (Table 3). Bulk samples were collected in a number of these facilities, analyzed for diacetyl, 2,3-pentanedione, and other VOCs, and compared to safety data sheets (44). The analysis revealed varying concentrations of diacetyl and 2,3-pentanedione in a flavoring sample and the presence of diacetyl in 81% and 2,3-pentanedione in 58% of samples.

Production and non-production workers in flavoring facilities had higher exposures and percentage of full-shift exposures above the NIOSH REL for diacetyl or 2,3-pentanedione than production workers in non-flavoring facilities or in cafés; café workers had higher exposures than the non-production workers in the non-flavoring facilities (Table 3). Full-shift exposures for flavoring/grinding operators (GM diacetyl range 34–26 ppb; GM 2,3-pentanedione range 38–22 ppb) measured in this study were lower than the levels measured for various job titles (GM diacetyl range 69–89 ppb; GM 2,3-pentanedione range 90–130 ppb) in the flavoring room of a flavored coffee production facility previously described by our group (10). Full-shift exposures for packaging worker in non-flavoring facilities (GM diacetyl 8.0 ppb; GM 2,3-pentanedione 4.4 ppb) and were comparable to those observed by McCoy et al. (45) for grinding (1.5 and 9.4 ppb diacetyl) and Pengelly et al. (46) (mean grinding/packing 7.4 ppb and 41 ppb diacetyl; mean 3.3 ppb and 22 ppb 2,3-pentanedione). In the flavored coffee facilities, the highest GM exposures to diacetyl were for flavoring, packaging, and grinding workers, while in the non-flavoring facilities, they were for packaging, QC, and general production workers; 2,3-pentanedione exposures followed a similar pattern with baristas included in the higher exposure group for non-flavoring. However, these average TWA concentrations do not inform us about short-term exposures, which were orders of magnitude higher and could be relevant to respiratory health, particularly when tasks are repeated multiple times per day. Moreover, average concentrations are not generally as useful as short-term task or source measurements in identifying options for exposure control measures. Given the diversity in facility layouts and process flows, full-shift exposures were likely influenced by multiple sources of exposure when workers were performing tasks in varying areas of the facilities.

The respiratory health risks associated with the full-shift exposures measured in these facilities are higher than NIOSH recommends. For example, geometric mean full-shift personal exposures ranged from 4.3 to 34 ppb diacetyl in flavored coffee facilities and 0.9 to 8.0 ppb in non-flavored coffee facilities (Table 5). After a 45-year working lifetime of continual exposure to 50 ppb diacetyl, NIOSH estimated that approximately 12 in 1,000 workers would develop reduced lung function (FEV1 below the lower limit of normal) [Table 5-29 in (18)]. NIOSH predicted approximately 1 in 1,000 workers exposed to diacetyl at 50 ppb would develop more severe lung function reduction [FEV1 below 60% predicted, Table 5-27 in (18)]. FEV1 below 60% predicted is defined as at least moderately severe by the American Thoracic Society (17). The respiratory health risks will change depending on an individual worker's exposure to diacetyl.

This study is the first to report personal task-based exposure estimates in coffee roasting facilities and cafés. Air samples were collected for short durations ranging from ~30 s to 86 min to effectively capture high exposures to emitted alpha-diketones. Flavoring coffee, grinding and packaging coffee were the most concerning short duration tasks for exposures to diacetyl and 2,3-pentanedione; flavoring was associated with highest exposures for 2,3-pentanedione, but not for diacetyl. In non-flavoring facilities, grinding and moving coffee had the highest task exposures to diacetyl and 2,3-pentanedione. Gaffney et al. (47) found grinding to be the greatest source of exposure in a roasting facility. In our study, silica gel sorbent tubes were effective at sampling for a few minutes because of a modification to the analytical method that enhanced sensitivity (30). GSDs were higher for some tasks compared to personal full-shift estimates because of inherent environmental variability in shorter term measurements (i.e., environmental variability is dampened in full-shift sampling because of a longer averaging interval). Short duration task exposures were generally over an order of magnitude higher than the full-shift exposures and provided important information on tasks that can be targeted for intervention.

We also collected instantaneous activity exposures from the workers' breathing zones during certain activities, and instantaneous source measurements at the emission source to inform instantaneous peak exposures for activities and at sources. As with short duration tasks, these instantaneous activities and source peak exposures may be important for respiratory health as well as in identifying contributions to emissions. We identified the activity of grinding and the source of ground coffee to be some of the greatest contributors to worker exposures to volatile emissions from unflavored coffee. The source and activity of flavoring coffee were also strong contributors to exposure especially for 2,3-pentanedione, a common diacetyl substitute. The instantaneous source measurements were much greater than the instantaneous activity exposures and provide critical information on options for controlling exposures at the source; information on activity exposures may be useful for planning administrative controls while implementing engineering controls.

Canister sampling was used for instantaneous grab sampling to complement sorbent tube sampling but could have been used for any sampling period. An added benefit of canister sampling was the collection of additional VOC analytes that allowed for quantification of ethanol and acetaldehyde among others. Measured ethanol concentrations are indicative of residual solvent in flavoring formulations. Acetaldehyde is an intermediate in flavoring manufacturing and classified by IARC as possibly carcinogenic to humans (Group 2B) (48) and by ACGIH^®^ as a suspected human carcinogen (A2) (37). Exposures to acetaldehyde were below the OSHA PEL of 200 ppm and less than the ACGIH^®^ TLV^®^ ceiling of 25 ppm, but acetaldehyde emissions during grinding and flavoring should be explored further using standard methods. The ACGIH^®^ TLV^®^ value was set based on eye and upper respiratory tract irritation.

Simultaneous exposure to multiple alpha-diketones as well as exposure to a complex mixture of VOCs, particulate and gaseous exposures occur during coffee processing. In this study, we created a mixture index to account for simultaneous exposure to diacetyl and 2,3-pentanedione using the ACGIH^®^ formula (37). OSHA uses a similar equation of summing the quotients of the components of the mixture to evaluate whether an exposure limit has been exceeded (49). We limited the components to two substances that have been associated with obliterative bronchiolitis and that have exposure limits. Our results show that most job groups in flavored coffee facilities had 100% of measurements above the mixture index, and for non-flavoring facilities only the Administrative job groups had <50% of measurements above the mixture index. To better represent workplace mixed exposures, future epidemiologic studies should consider using a mixed exposure metric or multipollutant model to address the effects of this complex exposure mixture on respiratory health.

In our assessments, diacetyl and 2,3-pentanedione background air concentrations increased over the workshift indicating a lack of adequate ventilation to keep concentrations to pre-shift levels. To address these potentially harmful levels of alpha-diketones, changes should be made according to the typical hierarchy of controls: eliminate/substitute, engineering controls, administrative controls, and personal protective equipment. This approach prioritizes actions by their likely effectiveness in reducing or removing hazards. In most cases, the preferred approach is to eliminate or substitute hazardous materials. Chemicals known to be hazardous should not be substituted with chemicals of unknown toxicity, which was the case with 2,3-pentanedione prematurely replacing diacetyl in some flavoring formulations. Elimination/substitution is not entirely feasible as diacetyl and 2,3-pentanedione exposures arise not only from the addition of flavorings, but are also generated when roasting coffee beans. Thus, installation of engineering controls should be considered to reduce exposures or shield workers.

Controlling emissions using local exhaust ventilation at sources, such as grinding machines and flavoring stations, might be the most effective means of reducing worker exposures to alpha-diketones. Local exhaust ventilation and enclosures that separate the roasted coffee or flavoring source from the worker should be designed and incorporated at grinding and flavoring areas. Isolating the coffee emission source from the workers by using loose-fitting lids on bins or silos of roasted coffee might reduce exposures by reducing emissions into the workspace, but care should be taken when opening the bins because peak exposures may occur. Isolation of the flavoring room or area from the main production space along with effective ventilation and isolation of the production space from the administrative or non-production space is essential for maintaining pollutant control. Note, however, that isolation of a source or process will increase worker exposures in or from the isolated areas if effort is not made to simultaneously control emissions in the isolated areas using ventilation. We have seen substantial reductions (one to three orders of magnitude) in diacetyl air concentrations by segregating processes and by using local exhaust ventilation at a microwave popcorn plant (14). General dilution ventilation is not recommended to control toxic chemical emissions because they are not effectively removed from the environment, just diluted and dispersed. A well-designed general ventilation system, however, might reduce air concentrations of toxic chemicals such as diacetyl and 2,3-pentanedione by providing outdoor air that is presumably contaminant-free and exhausting contaminants from the indoor air.

The American National Standards Institute (ANSI) and ASHRAE have developed consensus standards and guidelines for general dilution ventilation systems. ANSI/ASHRAE 62.1-2019 recommends outdoor air supply rates that take into account people-related sources as well as building-related sources. There are no specific recommendations in the standard for coffee roasting, packaging and flavoring facilities, or for coffee cafés. However, there are recommendations for similar spaces that can be used as a starting point for dilution ventilation systems. For instance, small to medium coffee production spaces could use the recommendation for sorting, packing, and light assembly areas. Those spaces should receive fresh, outdoor air at the rate of 7.5 cubic feet per minute (cfm)/person for people-related sources, and an additional 0.12 cfm for every square foot (cfm/ft^2^) of occupied space to account for building-related sources (50). Medium to large production areas could use the recommendation for manufacturing areas of 10 cfm/person plus 0.18 cfm/ft^2^. The recommendations for restaurant dining rooms, café/fast-food dining, and bars and cocktail lounges could be used for coffee cafés. They are recommended to be ventilated at 7.5 cfm/person plus 0.18 cfm/ft^2^ (50). Engineering controls should be designed and implemented by qualified ventilation engineers and companies. Process modification or automation to reduce the time workers spend around the emission source are further examples of engineering controls. Modifying work practices that require workers to place their heads near open containers of roasted coffee might reduce exposures. Automatic weighing and mixing of roasted coffee and flavoring of roasted coffee would also reduce exposures.

Administrative controls are next in the hierarchy after engineering controls. An effective administrative control is worker education on potential occupational hazards (e.g., diacetyl, 2,3-pentanedione, CO, CO_2_, green bean and roasted coffee dust) and respiratory health consequences of exposure.

Respiratory protection should be the last line of defense, but respirators might be needed as an interim control while permanent engineering and administrative controls can be implemented, and efficacy assessed. If respiratory protection is used, selection of the appropriate respirator should be guided by personal exposure sampling (51) and a written respiratory protection program should be implemented as required by the OSHA Respiratory Protection Standard (29 CFR 1910.134), including training, fit testing, medical evaluation, maintenance and use requirements.

### Limitations and Further Research

A potential limitation of the study is exposure misclassification during assignment of job groups in the production area as the administrative job titles were broad. Information obtained during the survey was used to assign these groups based on standardized sample data collection sheets and observations by the sampling team; thus, we expect this misclassification to be minimal. When an exposure is misclassified to an inappropriate job group, the group means and variance can be artificially increased or decreased. The effect of the misclassification will increase with decreasing group sample size. Another limitation of the study is the representativeness of the facilities evaluated and a potential for selection bias. As these investigations were initiated by facility owners or employees through the HHE program, it is not a random sample of facilities; a facility might not volunteer to participate if they have high exposures or if there are currently worker health concerns. While there is a possibility of selection bias, its effect on exposure is likely minimal. The exposure estimates for jobs and tasks reported here are within similar ranges to those reported in other published studies (45–47). A large number of samples were collected from numerous small to medium sized workplaces to characterize exposures to alpha-diketones associated with tasks, jobs, locations and sources at facilities that roast, grind and package coffee, and represents a valuable resource to estimate exposure for similar activities and workplace settings. Additionally, we could not balance the exposure groups, or the size of the facilities being tested as we had no control over the selection. This analysis did not include large facilities (i.e., >500 employees), where over 50% of employees in the coffee industry work. Most of the facilities in this study were small to medium size based on the total number of workers, which likely affected work processes, production volumes, and exposure levels. Thus, large facilities were not represented in this study and their exposures remain uncharacterized. Some facilities had segregation of production and non-production spaces. Finally, the exposure estimates should be interpreted carefully, especially the estimates of P95 for short-duration and instantaneous tasks, activities and sources due to the large variability (GSD) and censored data, combined with sometimes small sample size. Furthermore, the Bayesian analysis assumes that the priors selected were reasonable. While most priors were left vague to allow the data to drive the inference, we did restrict the GSDs in the repeated measures ANOVA in order to restrict possible GSDs to ranges typically seen in personal time-weighted averages. We also assume that measurements below the limit of detection follow similar trends as the observed measurements (52, 53). Additional assumptions associated with ANOVA include normality of errors, independence of individuals (or observations within a non-repeated measures ANOVA), and constant variances within- and between-workers. The P95 estimates also assume lognormality of exposures. In future analyses, we will assess determinants of exposures for full-shift TWA samples and task-based samples to further elucidate the mechanisms driving exposure concentrations in this industry.

## Conclusions

Obliterative bronchiolitis has previously been observed in the food and flavoring industries (14, 54, 55) and at two coffee facilities that flavored coffee (13, 26). Recently, obliterative bronchiolitis was reported in an individual in India who had worked for 20 years in a coffee facility that roasted and ground coffee; he quit after developing respiratory symptoms (56). Exposure assessments at 17 coffee roasting and packaging facilities revealed exposures to diacetyl above the REL in 95% and to 2,3-pentanedione in 77% of production samples in facilities that flavored coffee. The mixed exposure index for these two chemicals exceeded the mixture index among 96% of production samples in facilities that flavored coffee, 72% in non-flavored coffee facilities, and 67% in cafés. Grinding and flavoring coffee were the main tasks associated with elevated exposures. Controlling emissions at grinding machines and flavoring areas might be the most effective means of reducing worker exposures. Isolating higher exposure concentration areas (e.g., flavoring, grinding, and packaging areas) from the main production space and administrative or non-production spaces is essential for maintaining exposure control. Assessments of diacetyl and 2,3-pentanedione exposures in other coffee facilities is recommended because of the inherent variability in exposures among facilities caused by differences in facility design, workforce, processes, or work flows.

## Data Availability Statement

Due to restrictions imposed under the US Privacy Act and the limitations of what participants consented to, the data underlying the analyses presented, beyond what is provided in the paper, are confidential and not available to researchers outside the National Institute for Occupational Safety and Health (NIOSH). For more information about NIOSH's policy regarding sensitive data, see https://www.cdc.gov/niosh/ocas/datahandle.html. Requests to access the datasets should be directed to Ryan F. LeBouf, rlebouf@cdc.gov.

## Ethics Statement

The studies involving human participants were reviewed and approved by The NIOSH Institutional Review Board reviewed and approved this study (NIOSH Protocol 17-RHD-06XP). All participants provided their written informed consent to participate. The patients/participants provided their written informed consent to participate in this study.

## Author Contributions

RL, KC, RN, BB, AF, MS, SM, MD, RB, KF, JC-G, and MV contributed to conception and design of the study. AR and DB analyzed the silica gel tube and canister samples. NE and KF organized the database. RL, CG, NE, and MV performed the statistical analyses. RL wrote the first draft of the manuscript. All authors contributed to manuscript revision, read, and approved the submitted version.

## Conflict of Interest

The authors declare that the research was conducted in the absence of any commercial or financial relationships that could be construed as a potential conflict of interest.
