# Supplementary material for: Exposures and Emissions in Coffee Roasting Facilities and Cafés: Diacetyl, 2,3-Pentanedione, and Other Volatile Organic Compounds
Source: Front Public Health. 2020 Sep 18;8:561740. doi: 10.3389/fpubh.2020.561740 (PMC7531227; doi:10.3389/fpubh.2020.561740)
Supplement: Supplementary file 1 [file Table_1.DOCX]

Supplementary Material

# Supplementary Tables

Supplementary Table S1. Task descriptions at 17 sampled coffee roasting facilities and associated cafés

| **Task/Activity** | **Task/Activity Description** | **Where Sampled** |
| --- | --- | --- |
| Miscellaneous café tasks | - Taking orders at register - Filling coffee carafes - Cleaning - Making cappuccino - Making espresso - Making pour over - Steaming milk with steam wand - Pouring espresso shot - Melting chocolate with hot water | Cafés |
| Cleaning machines | Cleaning   - Grinders - Packaging machines - Flavoring mixers - Roasters | Flavored and non-flavored coffee facilities |
| Grinding coffee beans | - Grinding flavored and unflavored roasted coffee in various amounts from 8oz to 40lbs | Flavored and non-flavored coffee facilities |
| Maintenance of machines | - Adjustment and repair of packaging line and controls | Single non-flavored coffee facility |
| Miscellaneous production | - Scooping green beans - Moving pallets of coffee around the facility - Making labels - Loading green beans into roasters and hoppers - Moving green beans around facility | Flavored and non-flavored coffee facilities |
| Moving roasted beans or ground coffee | - Scooping roasted whole bean coffee into packaging machine - Pouring whole beans into buckets to hand blend - Pouring beans into storage bins - Moving silo of roasted coffee beans - Move supersack of roasted coffee - Mixing whole bean blend - Mixing and weighing whole beans in bins | Flavored and non-flavored coffee facilities |
| Packaging coffee | - Operating automatic packaging machines - Changing ribbon of bags on machines - Loading bags of coffee into boxes - Assembling boxes - Packing boxes onto pallets - Manually filling bags of coffee - Scooping and weighing coffee to be packaged | Flavored and non-flavored coffee facilities |
| Quality control | - Sizing green beans with sieves - Small batch (~10-15g) sample roasting and grinding - Cupping - Tasting - Making pour over coffee - Making espresso - Weighing coffee - Assessing color of roasted coffee | Flavored and non-flavored coffee facilities |
| Packaging rework | - Opening defective sealed bags of ground coffee by hand - Transferring ground coffee back to the packaging machine | Single flavored coffee facility |
| Roasting coffee beans | - Operating roasting machines - Transferring green beans to roaster - Listening for first crack - Emptying roaster into cooling drum or tray - Transferring roasted beans from cooling drum to storage or transfer vessels | Flavored and non-flavored coffee facilities |
| Pulling sample of beans during roasting | - Pulling samples of beans from roaster to check quality by smelling and samples for grinding and color assessment | Flavored and non-flavored coffee facilities |
| Flavoring coffee | - Weighing flavoring - Operating mixer for adding flavoring to roasted coffee beans - Adding flavoring to hopper for mixer | Flavored coffee facilities |

Supplementary Table S2. VOC emission source descriptions at 17 sampled coffee facilities and associated cafés

| **Source** | **Source Description** | **Where source was sampled** |
| --- | --- | --- |
| Café grinder | - Roasted coffee ground at café grinder - Ground coffee at café grinder | Cafés |
| Flavored coffee | - Flavoring whole bean - Transferring flavored ground coffee - At the flavoring blender discharge - At flavored whole bean hopper - Above flavoring hopper ground - Above flavored whole bean storage container | Flavored coffee facilities |
| Flavoring | - Transferring flavoring - Transferring flavoring inside bottle - Transferring above empty flavoring hopper | Flavored coffee facilities |
| Ground coffee | - During grinding or at the grinder with various amounts of coffee from 1 to >400 lbs - Grinding flavored and unflavored coffee - Transferring ground coffee - At ground transfer container | Flavored and non-flavored coffee facilities |
| Heat sealing bags | - Heat sealing of bags for coffee | Non-flavored coffee facilities |
| Miscellaneous café | - Frothed milk - Melted chocolate - Pour over coffee - Beverage preparation - Cold brew preparation vessel | Cafés |
| Miscellaneous QC | - Cupping - Espresso preparation - Pour over - Beverage - Sample waste container | Flavored and non-flavored coffee facilities |
| Packaging roasted coffee | - Automatic packaging machine (6 oz, 10 oz, 12 oz, 5 lb) - Transferring packaged coffee - Automatic packaging machine exhaust | Flavored and non-flavored coffee facilities |
| QC grinding | - Sample grinding - Sample grinder - Ground sample | Non-flavored coffee facilities |
| Roasted coffee | - Whole bean automatic blender - Hand blending whole bean - Hand packing whole bean - Transferring whole bean | Flavored and non-flavored coffee facilities |
| Roasted coffee in bag | - At or above roasted whole bean coffee in bags | Non-flavored coffee facilities |
| Roasted coffee in container | - Roasted whole bean coffee in storage containers | Non-flavored coffee facilities |
| Roaster cooling drum | - At or above roaster cooling drum | Non-flavored coffee facilities |
| Roaster door | - At roaster door | Non-flavored coffee facilities |
| Roasting | - Roasting - Transferring green beans - Roaster destoner - Cleaning roaster chaff exhaust | Non-flavored coffee facilities |
| Sample roaster | - At or above sample roaster | Non-flavored coffee facilities |

Supplementary Table S3. Work area descriptions at 17 sampled coffee facilities and associated cafés

| **Work Area** | **Area Description** |
| --- | --- |
| Administration area | Administrative area that is physically segregated by walls from production areas. This area is present in both flavoring and non-flavoring facilities. |
| Bakery/Café | Bakery, kitchen or café areas; may be attached to a production space or in a separate building. This area is only present in non-flavoring facilities and cafés. |
| Breakroom | An area where workers may congregate for refreshment or relax during breaks. This area is present in flavoring and non-flavoring facilities. |
| Flavoring area | Segregated area where flavoring is added to roasted coffee beans and grounds coffee. This area is only present in flavoring facilities. |
| Green bean storage area | Storage area for green beans. This area is present in flavoring and non-flavoring facilities. |
| Grinding area | Segregated area where roasted coffee beans are ground. This area is present in flavoring and non-flavoring facilities. |
| Outside-Café | Outside area of café facility for comparison to indoor area air concentrations. This area was only sampled outside cafés. |
| Outside-Production | Outside area of production facility for comparison to indoor area air concentrations. This area was sampled outside flavoring and non-flavoring facilities. |
| Packaging area | Segregated area where roasted coffee beans and ground coffee are packaged. This area is present in flavoring and non-flavoring facilities. |
| Production area | Main production space for facilities where areas were either segregated or not segregated (i.e., an open warehouse or garage). This area is present in flavoring and non-flavoring facilities. |
| Production storage area | Segregated area where production materials or finished products were stored. This area is present in flavoring and non-flavoring facilities. |
| Quality control area | Area where roasted coffee is tested for color, smell, taste, etc. This area is present in flavoring and non-flavoring facilities. |
| Roasting area | Area where green beans are roasted. This area is present in flavoring and non-flavoring facilities. |
| Shipping area | Products are shipped from this area. This area is only present in non-flavoring facilities. |

Supplementary Table S4. Job group descriptions at 17 sampled coffee facilities and associated cafés

| **Job Group** | **Job Description** |
| --- | --- |
| Administrative non-production worker | Administrative job (e.g., bookkeeping, chief executive officer, chief financial officer, customer service, director of operations, front office administration, graphic designer, green coffee buyer, manager owner, project coordinator sales, sales representative, sales and training, staff accountant, invoicing, inventory controller, etc.) that is not involved in production work. These jobs generally worked in administrative, non-production areas unless the facility was an open space with minimal or no segregation between production and non-production areas. This job is done in both flavoring and non-flavoring facilities. |
| Administrative production worker | Administrative job (e.g., assistant production manager, business manager, delivery driver, director of coffee/operations, inventory controller, label maker/production, line manager, materials manager, procurement specialist, production manager/supervisor, purchasing director, sales/customer service, shift supervisor, shipping/logistics/packaging, web design/order, web sales/marketing, purchasing administrator) sometimes requiring movement around the facility in production and non-production areas. This worker either worked in production areas or worked in a facility with minimal separation of production and non-production spaces. This job is done in both flavoring and non-flavoring facilities. |
| Barista | Takes customer orders, makes coffee drinks, and tends register. This job is only done in cafés. |
| Flavoring worker | Adds flavoring to roasted coffee. This job is only done in flavoring facilities. |
| Grinder operator | Grinds roasted coffee. This job is done in both flavoring and non-flavoring facilities. |
| Other café worker | All other jobs excluding barista (e.g., events coordinator, café quality control/education manager, shop manager, baker, director of education, director of kitchen operations. This job is only done in non-flavoring facilities and cafés. |
| Packaging worker | Packages roasted whole bean and ground coffee. This job is done in both flavoring and non-flavoring facilities. |
| Production worker | General duties related to the overall production of roasted coffee, which may include general labor, supervisors, or managers. This job is done in both flavoring and non-flavoring facilities. |
| Production support worker | Aides in the production of roasted coffee but does not directly produce roasted coffee (e.g., maintenance mechanic, operations specialist, service department workers, plant engineer, forklift operator). This job is done in both flavoring and non-flavoring facilities. |
| Quality control worker | Conducts quality control tests of coffee including cupping, tasting, and color. This job is done in both flavoring and non-flavoring facilities. |
| Roaster operator | Operates roasting machine or roaster. This job is done in both flavoring and non-flavoring facilities. |

Supplementary Table S5. Median analyte limits of detection (LODs) and limits of quantitation (LOQs) using evacuated canisters (NMAM 3900)

| **Analyte** | **VOC canister source median LOD (ppb)** | **VOC canister source median LOQ (ppb)** | **VOC canister task median LOD (ppb)** | **VOC canister task median LOQ (ppb)** |
| --- | --- | --- | --- | --- |
| 2,3-Hexanedione | 1.4 | 4.7 | 1.5 | 5.0 |
| 2,3-Pentanedione | 0.8 | 2.7 | 0.8 | 2.7 |
| Acetaldehyde | 0.6 | 2.0 | 0.8 | 2.7 |
| Acetone | 1.7 | 5.7 | 2.1 | 7.0 |
| Acetonitrile | 0.7 | 2.3 | 0.8 | 2.7 |
| α-Pinene | 0.9 | 3.0 | 1.0 | 3.3 |
| Benzene | 0.6 | 2.0 | 0.6 | 2.0 |
| Chloroform | 0.6 | 2.0 | 0.6 | 2.0 |
| Diacetyl | 0.6 | 2.0 | 0.6 | 2.0 |
| *d*-Limonene | 1.5 | 5.0 | 1.7 | 5.7 |
| Ethanol | 1.4 | 4.7 | 1.7 | 5.7 |
| Ethylbenzene | 0.8 | 2.7 | 0.8 | 2.7 |
| Isopropyl alcohol | 1.0 | 3.3 | 1.2 | 4.0 |
| *m,p*-Xylene | 0.6 | 2.0 | 0.7 | 2.3 |
| Methyl methacrylate | 0.5 | 1.7 | 0.6 | 2.0 |
| Methylene chloride | 0.5 | 1.7 | 0.6 | 2.0 |
| n-Hexane | 0.9 | 3.0 | 1.0 | 3.3 |
| *o*-Xylene | 0.6 | 2.0 | 0.6 | 2.0 |
| Styrene | 1.0 | 3.3 | 1.1 | 3.7 |
| Toluene | 0.7 | 2.3 | 0.8 | 2.7 |

Supplementary Table S6. Job group personal TWA exposures to acetoin and 2,3-hexanedione using modified OSHA Methods 1013/1016

|  |  |  | **Acetoin** | | | | **2,3-Hexanedione** | | | |
| --- | --- | --- | --- | --- | --- | --- | --- | --- | --- | --- |
| **Job Group** | **N** | **k** | **GM (ppb)** | **GSD** | **P95 or max* (ppb)** | **%BDL** | **GM (ppb)** | **GSD** | **P95 or max* (ppb)** | **%BDL** |
| **NON-FLAVOR** |  |  |  |  |  |  |  |  |  |  |
| Administrative non-production worker | 38 | 23 | – | – | 4.6* | 92 | – | – | – | 100 |
| Administrative production worker | 53 | 25 | 0.6 | 2.4 | 2.8 | 72 | – | – | 0.9* | 98 |
| Barista | 14 | 13 | – | – | 2.7* | 86 | – | – | – | 100 |
| Grinder operator | 3 | 3 | – | – | 2.0* | 67 | – | – | – | 100 |
| Other café worker | 7 | 7 | – | – | 3.6* | 71 | – | – | 0.5* | 71 |
| Packaging worker | 80 | 41 | 1.1 | 1.9 | 3.1 | 60 | – | – | 1.3* | 95 |
| Production worker | 36 | 24 | 0.7 | 3.5 | 5.6 | 67 | 0.1 | 4.0 | 1.1 | 78 |
| Production support worker | 9 | 4 | – | – | 2.3* | 67 | – | – | 0.5* | 89 |
| Quality control worker | 15 | 5 | 1.1 | 1.9 | 3.1 | 40 | – | – | 0.5* | 93 |
| Roaster operator | 63 | 34 | 1.1 | 2.1 | 3.7 | 60 | 0.05 | 4.7 | 0.6 | 90 |
| **FLAVOR** |  |  |  |  |  |  |  |  |  |  |
| Administrative non-production worker | 7 | 5 | 5.3 | 25 | 986 | 29 | – | – | – | 100 |
| Administrative production worker | 6 | 3 | 8.8 | 4.4 | 99 | 0 | – | – | 0.5* | 83 |
| Flavoring worker | 7 | 4 | 163 | 8.7 | 5,622 | 0 | – | – | 11* | 43 |
| Grinder operator | 5 | 2 | 12 | 2.3 | 47 | 0 | – | – | 2.0* | 40 |
| Packaging worker | 44 | 27 | 43 | 3.1 | 276 | 0 | 0.2 | 3.0 | 1.3 | 73 |
| Production worker | 5 | 3 | – | – | 3.5* | 60 | – | – | – | 100 |
| Production support worker | 3 | 2 | – | – | 166* | 0 | – | – | – | 100 |
| Quality control worker | 4 | 4 | – | – | 117* | 0 | – | – | – | 100 |
| Roaster operator | 17 | 7 | 9.7 | 11 | 467 | 12 | – | – | 0.5* | 94 |

TWA=time-weighted average; N=number of samples; k=number of workers; GM=geometric mean; ppb=parts per billion; GSD=geometric standard deviation; P95=95^th^ percentile; %BDL=percent samples below the limit of detection; max*=maximum presented when <5 measurements were above the detection limit; – =not enough measurements above the detection limit to obtain an estimate.

Supplementary Table S7. Area TWA concentrations of acetoin and 2,3-hexanedione using modified OSHA Methods 1013/1016

|  |  | **Acetoin** | | | | **2,3-Hexanedione** | | | |
| --- | --- | --- | --- | --- | --- | --- | --- | --- | --- |
| **Area** | **N** | **GM (ppb)** | **GSD** | **P95 or max* (ppb)** | **%BDL** | **GM (ppb)** | **GSD** | **P95 or max* (ppb)** | **%BDL** |
| **NON-FLAVOR** |  |  |  |  |  |  |  |  |  |
| Administration area | 63 | – | – | 2.4* | 94 | – | – | – | 100 |
| Bakery/Café | 54 | 0.3 | 3.8 | 2.5 | 89 | – | – | 0.9* | 98 |
| Breakroom | 9 | – | – | – | 100 | – | – | – | 100 |
| Green bean storage area | 7 | – | – | – | 100 | – | – | – | 100 |
| Grinding area | 40 | 2.4 | 2.7 | 12.5 | 40 | 0.3 | 3.2 | 1.9 | 63 |
| Packaging area | 103 | 1.5 | 1.9 | 4.2 | 39 | 0.04 | 4.9 | 0.5 | 93 |
| Production area | 102 | 0.8 | 1.9 | 2.2 | 70 | – | – | 0.8* | 98 |
| Production storage area | 25 | 1.3 | 2.5 | 5.9 | 64 | – | – | 0.9* | 88 |
| Quality control area | 20 | 0.7 | 2.0 | 2.2 | 70 | – | – | – | 100 |
| Roasting area | 72 | 1.6 | 1.9 | 4.8 | 49 | – | – | 1.2* | 94 |
| Shipping area | 9 | – | – | 3.2* | 78 | – | – | – | 100 |
| **FLAVOR** |  |  |  |  |  |  |  |  |  |
| Administration area | 21 | 5.4 | 9.2 | 205 | 29 | – | – | 0.5* | 90 |
| Breakroom | 7 | 18 | 4.5 | 206 | 0 | – | – | – | 100 |
| Flavoring area | 19 | 304 | 8.1 | 9,440 | 0 | 0.1 | 25 | 16 | 68 |
| Green bean storage area | 12 | 10 | 6.0 | 195 | 0 | – | – | – | 100 |
| Grinding area | 26 | 20 | 6.3 | 402 | 7.7 | 0.2 | 3.2 | 1.4 | 69 |
| Packaging area | 87 | 37 | 3.8 | 328 | 2.3 | 0.1 | 4.0 | 0.9 | 89 |
| Production area | 12 | 19 | 4.4 | 217 | 0 | – | – | 0.9* | 83 |
| Production storage area | 35 | 15 | 9.8 | 652 | 17 | – | – | 1.0* | 97 |
| Quality control area | 7 | 14 | 4.5 | 161 | 0 | – | – | – | 100 |
| Roasting area | 30 | 10 | 9.1 | 374 | 13 | – | – | 0.9* | 93 |

TWA=time-weighted average; N=number of samples; GM=geometric mean; ppb=parts per billion; GSD=geometric standard deviation; P95=95^th^ percentile; %BDL=percent samples below the limit of detection; max*=maximum presented when <5 measurements were above the detection limit; – =not enough samples above the detection limit to obtain an estimate

Supplementary Table S8. Personal task exposures to acetoin and 2,3-hexanedione using modified OSHA Methods 1013/1016

|  | |  | **Acetoin** | | | | **2,3-Hexanedione** | | | |
| --- | --- | --- | --- | --- | --- | --- | --- | --- | --- | --- |
| **Task** | **N** | **k** | **GM (ppb)** | **GSD** | **P95 or max* (ppb)** | **%BDL** | **GM (ppb)** | **GSD** | **P95 or max* (ppb)** | **%BDL** |
| **NON-FLAVOR** |  |  |  |  |  |  |  |  |  |  |
| Miscellaneous café tasks | 10 | 6 | – | – | – | 100 | – | – | – | 100 |
| Cleaning machines | 9 | 6 | – | – | 4.6* | 78 | – | – | – | 100 |
| Grinding coffee beans | 58 | 25 | 3.6 | 3.0 | 22 | 60 | 0.5 | 2.8 | 3.0 | 78 |
| Maintenance of machines | 5 | 1 | – | – | – | 100 | – | – | – | 100 |
| Miscellaneous production | 9 | 5 | – | – | – | 100 | – | – | – | 100 |
| Moving roasted beans or ground coffee | 10 | 6 | – | – | – | 100 | – | – | 2.0* | 90 |
| Packaging coffee | 153 | 56 | 0.4 | 4.3 | 4.6 | 90 | 0.1 | 4.8 | 0.8 | 92 |
| Quality control | 40 | 9 | – | – | 4.4* | 98 | – | – | – | 100 |
| Packaging rework | 4 | 2 | – | – | 9.8* | 25 | – | – | 1.7* | 25 |
| Roasting coffee beans | 152 | 27 | 0.6 | 3.2 | 4.0 | 91 | – | – | 1.4* | 99 |
| **FLAVOR** |  |  |  |  |  |  |  |  |  |  |
| Cleaning machines | 27 | 12 | 24 | 9.4 | 884 | 15 | – | – | – | 100 |
| Flavoring coffee | 15 | 5 | 20 | 46 | 8,969 | 27 | – | – | 63* | 73 |
| Grinding coffee beans | 19 | 9 | 8.2 | 14 | 622 | 21 | 0.2 | 14.5 | 12 | 74 |
| Miscellaneous production | 3 | 3 | – | – | 16* | 67 | – | – | – | 100 |
| Moving roasted beans or ground coffee | 3 | 3 | – | – | 11* | 67 | – | – | – | 100 |
| Packaging coffee | 46 | 18 | 29 | 5.1 | 408 | 16 | – | – | 2.7* | 91 |
| Roasting coffee beans | 43 | 8 | 2.1 | 17 | 195 | 56 | – | – | – | 100 |

N=number of samples; k=number of workers; GM=geometric mean; ppb=parts per billion; GSD=geometric standard deviation; P95=95^th^ percentile; %BDL=percent samples below the limit of detection; max*=maximum presented when <5 measurements were above the detection limit; – =not enough measurements above the detection limit to obtain an estimate.

Supplementary Table S9. Personal instantaneous activity exposures to additional VOCs using evacuated canisters (NMAM 3900)

| **Analyte** | **Activity** | **N** | **GM (ppb)** | **GSD** | **P95 or max* (ppb)** | **%BDL** |
| --- | --- | --- | --- | --- | --- | --- |
| **NON-FLAVOR** |  |  |  |  |  |  |
| 2,3-Hexanedione | Grinding coffee beans | 67 | 1.2 | 4.5 | 15 | 46 |
| 2,3-Hexanedione | Miscellaneous café tasks | 6 | – | – | 6.5* | 33 |
| 2,3-Hexanedione | Moving roasted beans or ground coffee | 59 | 0.5 | 6.5 | 11 | 61 |
| 2,3-Hexanedione | Packaging coffee | 32 | 1.1 | 2.1 | 3.9 | 41 |
| 2,3-Hexanedione | Pulling sample of beans during roasting | 14 | – | – | 2.3* | 79 |
| 2,3-Hexanedione | QC | 20 | 2.4 | 2.1 | 8.4 | 10 |
| 2,3-Hexanedione | Roasting coffee beans | 16 | 1.2 | 3.1 | 7.4 | 44 |
| Acetaldehyde | Grinding coffee beans | 67 | 42 | 9 | 1,536 | 7.5 |
| Acetaldehyde | Miscellaneous café tasks | 6 | 37 | 1.3 | 55 | 0 |
| Acetaldehyde | Moving roasted beans or ground coffee | 59 | 6.7 | 25 | 1,296 | 29 |
| Acetaldehyde | Packaging coffee | 32 | 23 | 7.3 | 609 | 13 |
| Acetaldehyde | Pulling sample of beans during roasting | 14 | 20 | 3.6 | 161 | 7.1 |
| Acetaldehyde | QC | 20 | 59 | 1.7 | 139 | 0 |
| Acetaldehyde | Roasting coffee beans | 16 | 4.6 | 21 | 680 | 31 |
| Acetone | Grinding coffee beans | 67 | 89 | 4.9 | 1,204 | 3.0 |
| Acetone | Miscellaneous café tasks | 6 | 20 | 9.9 | 829 | 17 |
| Acetone | Moving roasted beans or ground coffee | 59 | 35 | 5.8 | 624 | 3.4 |
| Acetone | Packaging coffee | 32 | 59 | 2.1 | 198 | 0 |
| Acetone | Pulling sample of beans during roasting | 14 | 50 | 3.2 | 331 | 0 |
| Acetone | QC | 20 | 97 | 1.5 | 186 | 0 |
| Acetone | Roasting coffee beans | 16 | 26 | 4.4 | 290 | 6.3 |
| α-Pinene | Grinding coffee beans | 67 | 1.0 | 1.7 | 2.4 | 31 |
| α-Pinene | Miscellaneous café tasks | 6 | 2.3 | 1.3 | 3.6 | 0 |
| α-Pinene | Moving roasted beans or ground coffee | 59 | 0.7 | 2 | 2.4 | 42 |
| α-Pinene | Packaging coffee | 32 | 0.8 | 1.9 | 2.4 | 38 |
| α-Pinene | Pulling sample of beans during roasting | 14 | 0.3 | 4.7 | 4.1 | 64 |
| α-Pinene | QC | 20 | 1.1 | 1.4 | 1.9 | 0 |
| α-Pinene | Roasting coffee beans | 16 | 0.7 | 2.0 | 2.3 | 50 |
| Benzene | Grinding coffee beans | 67 | 0.5 | 3.3 | 3.7 | 40 |
| Benzene | Miscellaneous café tasks | 6 | 0.7 | 1.8 | 1.8 | 0 |
| Benzene | Moving roasted beans or ground coffee | 59 | 0.3 | 3.9 | 3.2 | 49 |
| Benzene | Packaging coffee | 32 | 0.4 | 4.2 | 4.1 | 47 |
| Benzene | Pulling sample of beans during roasting | 14 | – | – | 3.9* | 79 |
| Benzene | QC | 20 | 0.5 | 2.7 | 2.6 | 25 |
| Benzene | Roasting coffee beans | 16 | 0.5 | 6 | 8.4 | 50 |
| *d*-Limonene | Grinding coffee beans | 67 | 2.2 | 3.5 | 18 | 28 |
| *d*-Limonene | Miscellaneous café tasks | 6 | 3.5 | 1.5 | 6.8 | 0 |
| *d*-Limonene | Moving roasted beans or ground coffee | 59 | 1.7 | 4.9 | 23 | 39 |
| *d*-Limonene | Packaging coffee | 32 | 0.4 | 7.4 | 10 | 69 |
| *d*-Limonene | Pulling sample of beans during roasting | 14 | 3.4 | 4.6 | 41 | 14 |
| *d*-Limonene | QC | 20 | 16 | 3.3 | 110 | 0 |
| *d*-Limonene | Roasting coffee beans | 16 | 1.9 | 2.2 | 7.2 | 25 |
| Ethanol | Grinding coffee beans | 67 | 40 | 3.4 | 309 | 1.5 |
| Ethanol | Miscellaneous café tasks | 6 | 233 | 1.3 | 367 | 0 |
| Ethanol | Moving roasted beans or ground coffee | 59 | 33 | 3.4 | 248 | 1.7 |
| Ethanol | Packaging coffee | 32 | 57 | 3.1 | 367 | 0 |
| Ethanol | Pulling sample of beans during roasting | 14 | 17 | 4.5 | 205 | 0 |
| Ethanol | QC | 20 | 21 | 2.4 | 88 | 0 |
| Ethanol | Roasting coffee beans | 16 | 39 | 3.0 | 239 | 0 |
| Ethylbenzene | Grinding coffee beans | 67 | 0.5 | 2.8 | 2.7 | 57 |
| Ethylbenzene | Miscellaneous café tasks | 6 | 1.3 | 1.6 | 2.8 | 0 |
| Ethylbenzene | Moving roasted beans or ground coffee | 59 | 0.4 | 3.2 | 2.4 | 64 |
| Ethylbenzene | Packaging coffee | 32 | 0.2 | 3.5 | 1.3 | 81 |
| Ethylbenzene | Pulling sample of beans during roasting | 14 | – | – | – | 100 |
| Ethylbenzene | QC | 20 | 0.6 | 2.4 | 2.4 | 45 |
| Ethylbenzene | Roasting coffee beans | 16 | 0.4 | 6.5 | 7.6 | 63 |
| Isopropyl alcohol | Grinding coffee beans | 67 | 21 | 8.2 | 666 | 6.0 |
| Isopropyl alcohol | Miscellaneous café tasks | 6 | 9.3 | 1.8 | 25 | 0 |
| Isopropyl alcohol | Moving roasted beans or ground coffee | 59 | 8.4 | 6.1 | 167 | 6.8 |
| Isopropyl alcohol | Packaging coffee | 32 | 5.5 | 3.7 | 46 | 9.4 |
| Isopropyl alcohol | Pulling sample of beans during roasting | 14 | 15 | 5.6 | 256 | 0 |
| Isopropyl alcohol | QC | 20 | 13 | 1.5 | 27 | 0 |
| Isopropyl alcohol | Roasting coffee beans | 16 | 8.0 | 3.5 | 63 | 0 |
| *m,p*-Xylene | Grinding coffee beans | 67 | 0.9 | 2.7 | 4.4 | 28 |
| *m,p*-Xylene | Miscellaneous café tasks | 6 | 2.3 | 2.0 | 7 | 0 |
| *m,p*-Xylene | Moving roasted beans or ground coffee | 59 | 0.7 | 2.9 | 3.8 | 34 |
| *m,p*-Xylene | Packaging coffee | 32 | 0.8 | 2.3 | 3 | 19 |
| *m,p*-Xylene | Pulling sample of beans during roasting | 14 | – | – | 0.9* | 71 |
| *m,p*-Xylene | QC | 20 | 1.0 | 2.2 | 3.7 | 5.0 |
| *m,p*-Xylene | Roasting coffee beans | 16 | 0.8 | 6.9 | 20 | 38 |
| *o*-Xylene | Grinding coffee beans | 67 | 0.4 | 2.9 | 2.3 | 54 |
| *o*-Xylene | Miscellaneous café tasks | 6 | 1.1 | 1.8 | 2.9 | 0 |
| *o*-Xylene | Moving roasted beans or ground coffee | 59 | 0.3 | 3.2 | 1.9 | 61 |
| *o*-Xylene | Packaging coffee | 32 | 0.4 | 2.1 | 1.3 | 53 |
| *o*-Xylene | Pulling sample of beans during roasting | 14 | – | – | 0.4* | 93 |
| *o*-Xylene | QC | 20 | 0.6 | 2.1 | 1.8 | 25 |
| *o*-Xylene | Roasting coffee beans | 16 | 0.2 | 9.7 | 9.4 | 63 |
| Styrene | Grinding coffee beans | 67 | 0.4 | 2.9 | 2.2 | 75 |
| Styrene | Miscellaneous café tasks | 6 | 1.9 | 3.4 | 14 | 17 |
| Styrene | Moving roasted beans or ground coffee | 59 | 0.1 | 5.4 | 1.1 | 90 |
| Styrene | Packaging coffee | 32 | – | – | 1.1* | 94 |
| Styrene | Pulling sample of beans during roasting | 14 | – | – | – | 100 |
| Styrene | QC | 20 | – | – | 1.1* | 95 |
| Styrene | Roasting coffee beans | 16 | – | – | 1.2* | 88 |
| Toluene | Grinding coffee beans | 67 | 1.6 | 2.6 | 7.9 | 16 |
| Toluene | Miscellaneous café tasks | 6 | 4.7 | 1.5 | 9.5 | 0 |
| Toluene | Moving roasted beans or ground coffee | 59 | 1.1 | 3.5 | 8.5 | 27 |
| Toluene | Packaging coffee | 32 | 1.3 | 2.5 | 5.8 | 13 |
| Toluene | Pulling sample of beans during roasting | 14 | 0.9 | 2.3 | 3.5 | 29 |
| Toluene | QC | 20 | 2.9 | 1.9 | 8.2 | 0 |
| Toluene | Roasting coffee beans | 16 | 1.2 | 3.6 | 9.6 | 25 |
| **FLAVOR** |  |  |  |  |  |  |
| 2,3-Hexanedione | Flavoring coffee | 16 | 0.8 | 33 | 238 | 50 |
| 2,3-Hexanedione | Grinding coffee beans | 26 | 2.3 | 4.0 | 22 | 23 |
| 2,3-Hexanedione | Moving roasted beans or ground coffee | 11 | 2.8 | 1.4 | 4.8 | 9.1 |
| 2,3-Hexanedione | Packaging coffee | 7 | 4.5 | 2.3 | 18 | 0 |
| 2,3-Hexanedione | Pulling sample of beans during roasting | 6 | – | – | 8.4* | 50 |
| 2,3-Hexanedione | QC | 3 | – | – | 2.6* | 0 |
| 2,3-Hexanedione | Roasting coffee beans | 13 | 0.6 | 3.7 | 4.7 | 54 |
| Acetaldehyde | Flavoring coffee | 16 | 156 | 8.1 | 4,846 | 0 |
| Acetaldehyde | Grinding coffee beans | 26 | 203 | 2.8 | 1,074 | 0 |
| Acetaldehyde | Moving roasted beans or ground coffee | 11 | 161 | 1.5 | 308 | 0 |
| Acetaldehyde | Packaging coffee | 7 | 149 | 2.3 | 580 | 0 |
| Acetaldehyde | Pulling sample of beans during roasting | 6 | 84 | 3.2 | 549 | 0 |
| Acetaldehyde | QC | 3 | – | – | 121* | 0 |
| Acetaldehyde | Roasting coffee beans | 13 | 34 | 2.8 | 183 | 0 |
| Acetone | Flavoring coffee | 16 | 118 | 16 | 10,792 | 13 |
| Acetone | Grinding coffee beans | 26 | 196 | 4 | 1,934 | 3.8 |
| Acetone | Moving roasted beans or ground coffee | 11 | 173 | 1.7 | 414 | 0 |
| Acetone | Packaging coffee | 7 | 330 | 3.5 | 2,599 | 0 |
| Acetone | Pulling sample of beans during roasting | 6 | 84 | 3.2 | 572 | 0 |
| Acetone | QC | 3 | – | – | 74* | 0 |
| Acetone | Roasting coffee beans | 13 | 189 | 3.4 | 1,381 | 0 |
| α-Pinene | Flavoring coffee | 16 | 0.5 | 5.9 | 9 | 69 |
| α-Pinene | Grinding coffee beans | 26 | 1.6 | 2.1 | 5.6 | 31 |
| α-Pinene | Moving roasted beans or ground coffee | 11 | 3.0 | 1.2 | 3.8 | 9.1 |
| α-Pinene | Packaging coffee | 7 | 1.2 | 4.3 | 13 | 29 |
| α-Pinene | Pulling sample of beans during roasting | 6 | – | – | 2.7* | 67 |
| α-Pinene | QC | 3 | – | – | 2.4* | 33 |
| α-Pinene | Roasting coffee beans | 13 | – | – | 2.8* | 69 |
| Benzene | Flavoring coffee | 16 | 0.5 | 7.8 | 13 | 56 |
| Benzene | Grinding coffee beans | 26 | 0.1 | 11 | 6.9 | 69 |
| Benzene | Moving roasted beans or ground coffee | 11 | – | – | 2.4* | 91 |
| Benzene | Packaging coffee | 7 | – | – | 4.8* | 57 |
| Benzene | Pulling sample of beans during roasting | 6 | – | – | 6.8* | 83 |
| Benzene | QC | 3 | – | – | – | 100 |
| Benzene | Roasting coffee beans | 13 | – | – | 0.8* | 69 |
| *d*-Limonene | Flavoring coffee | 16 | 2.1 | 3.5 | 17 | 44 |
| *d*-Limonene | Grinding coffee beans | 26 | 2.7 | 2.7 | 14 | 27 |
| *d*-Limonene | Moving roasted beans or ground coffee | 11 | 2.3 | 4.8 | 30 | 27 |
| *d*-Limonene | Packaging coffee | 7 | 1.5 | 1.4 | 2.5 | 0 |
| *d*-Limonene | Pulling sample of beans during roasting | 6 | – | – | 14* | 33 |
| *d*-Limonene | QC | 3 | – | – | 9.8* | 0 |
| *d*-Limonene | Roasting coffee beans | 13 | – | – | 5.2* | 69 |
| Ethanol | Flavoring coffee | 16 | 8,765 | 8.0 | 263,320 | 0 |
| Ethanol | Grinding coffee beans | 26 | 195 | 6.4 | 4153 | 3.8 |
| Ethanol | Moving roasted beans or ground coffee | 11 | 333 | 1.8 | 880 | 0 |
| Ethanol | Packaging coffee | 7 | 244 | 2.5 | 1,076 | 0 |
| Ethanol | Pulling sample of beans during roasting | 6 | 283 | 4.1 | 2,814 | 0 |
| Ethanol | QC | 3 | – | – | 172* | 0 |
| Ethanol | Roasting coffee beans | 13 | 76 | 2.1 | 265 | 0 |
| Ethylbenzene | Flavoring coffee | 16 | 0.2 | 12 | 10 | 69 |
| Ethylbenzene | Grinding coffee beans | 26 | 0.3 | 3.7 | 2.2 | 73 |
| Ethylbenzene | Moving roasted beans or ground coffee | 11 | – | – | 1.5* | 82 |
| Ethylbenzene | Packaging coffee | 7 | 0.7 | 1.5 | 1.5 | 14 |
| Ethylbenzene | Pulling sample of beans during roasting | 6 | – | – | 2.0* | 67 |
| Ethylbenzene | QC | 3 | – | – | – | 100 |
| Ethylbenzene | Roasting coffee beans | 13 | – | – | 0.7* | 85 |
| Isopropyl alcohol | Flavoring coffee | 16 | 0.7 | 202 | 3,520 | 69 |
| Isopropyl alcohol | Grinding coffee beans | 26 | 11 | 19 | 1,324 | 35 |
| Isopropyl alcohol | Moving roasted beans or ground coffee | 11 | 86 | 3.1 | 549 | 9.1 |
| Isopropyl alcohol | Packaging coffee | 7 | 69 | 4.2 | 728 | 0 |
| Isopropyl alcohol | Pulling sample of beans during roasting | 6 | 22 | 12 | 1,198 | 17 |
| Isopropyl alcohol | QC | 3 | – | – | 84* | 0 |
| Isopropyl alcohol | Roasting coffee beans | 13 | 1.7 | 24 | 291 | 39 |
| *m,p*-Xylene | Flavoring coffee | 16 | 0.5 | 7.0 | 11 | 63 |
| *m,p*-Xylene | Grinding coffee beans | 26 | 0.8 | 3.1 | 4.9 | 35 |
| *m,p*-Xylene | Moving roasted beans or ground coffee | 11 | 0.7 | 2.6 | 3.4 | 36 |
| *m,p*-Xylene | Packaging coffee | 7 | 1.0 | 1.9 | 2.9 | 14 |
| *m,p*-Xylene | Pulling sample of beans during roasting | 6 | – | – | 2.8* | 67 |
| *m,p*-Xylene | QC | 3 | – | – | – | 100 |
| *m,p*-Xylene | Roasting coffee beans | 13 | 0.3 | 3.6 | 2.5 | 62 |
| *o*-Xylene | Flavoring coffee | 16 | 0.4 | 6.8 | 8.4 | 63 |
| *o*-Xylene | Grinding coffee beans | 26 | 0.4 | 2.8 | 2.1 | 58 |
| *o*-Xylene | Moving roasted beans or ground coffee | 11 | 0.4 | 3.3 | 2.6 | 55 |
| *o*-Xylene | Packaging coffee | 7 | 0.6 | 1.7 | 1.3 | 14 |
| *o*-Xylene | Pulling sample of beans during roasting | 6 | – | – | 1.8* | 67 |
| *o*-Xylene | QC | 3 | – | – | – | 100 |
| *o*-Xylene | Roasting coffee beans | 13 | – | – | 0.7* | 69 |
| Styrene | Flavoring coffee | 16 | 2.5 | 9.0 | 90 | 50 |
| Styrene | Grinding coffee beans | 26 | 0.2 | 6.9 | 3.6 | 81 |
| Styrene | Moving roasted beans or ground coffee | 11 | – | – | – | 100 |
| Styrene | Packaging coffee | 7 | – | – | 1.3* | 71 |
| Styrene | Pulling sample of beans during roasting | 6 | – | – | 4.4* | 67 |
| Styrene | QC | 3 | – | – | – | 100 |
| Styrene | Roasting coffee beans | 13 | – | – | 12* | 77 |
| Toluene | Flavoring coffee | 16 | 2.3 | 3.2 | 16 | 19 |
| Toluene | Grinding coffee beans | 26 | 1.5 | 2.2 | 5.5 | 7.7 |
| Toluene | Moving roasted beans or ground coffee | 11 | 1.3 | 2.2 | 4.7 | 0 |
| Toluene | Packaging coffee | 7 | 1.7 | 2.1 | 5.8 | 0 |
| Toluene | Pulling sample of beans during roasting | 6 | – | – | 2.8* | 33 |
| Toluene | QC | 3 | – | – | 0.8* | 0 |
| Toluene | Roasting coffee beans | 13 | 0.9 | 2.7 | 4.5 | 23 |

Note: Acetonitrile, chloroform, methyl methacrylate, methylene chloride, and n-hexane were not detected in >75% of samples; N=number of samples; GM=geometric mean; ppb=parts per billion; GSD=geometric standard deviation; P95=95^th^ percentile; %BDL=percent samples below the limit of detection; max*=maximum presented when <5 measurements were above the detection limit; – =not enough samples above the detection limit to obtain an estimate

Supplementary Table S10. Area instantaneous source concentrations of additional VOCs using evacuated canisters (NMAM 3900)

| **Analyte** | **Source** | **N** | **GM (ppb)** | **GSD** | **P95 or max* (ppb)** | **%BDL** |
| --- | --- | --- | --- | --- | --- | --- |
| **NON-FLAVOR** |  |  |  |  |  |  |
| 2,3-Hexanedione | Café grinder | 7 | 8.8 | 3.1 | 55 | 0 |
| 2,3-Hexanedione | Ground coffee | 52 | 11 | 13 | 741 | 21 |
| 2,3-Hexanedione | Heat sealing bags | 3 | – | – | 4.0* | 33 |
| 2,3-Hexanedione | Miscellaneous QC | 11 | 3.1 | 2.8 | 16 | 9.1 |
| 2,3-Hexanedione | Miscellaneous café | 7 | – | – | 8.1* | 43 |
| 2,3-Hexanedione | Packaging roasted coffee | 18 | 0.9 | 6.3 | 19 | 50 |
| 2,3-Hexanedione | QC grinding | 9 | 5.4 | 2.3 | 22 | 0 |
| 2,3-Hexanedione | Roasted coffee | 54 | 1.3 | 4.8 | 17 | 37 |
| 2,3-Hexanedione | Roasted coffee in bag | 5 | – | – | 54* | 20 |
| 2,3-Hexanedione | Roasted coffee in container | 53 | 8.4 | 7.0 | 203 | 15 |
| 2,3-Hexanedione | Roaster cooling drum | 12 | 1.0 | 4.1 | 10 | 42 |
| 2,3-Hexanedione | Roaster door | 10 | – | – | 76* | 70 |
| 2,3-Hexanedione | Roasting | 12 | 1.8 | 4.3 | 20 | 25 |
| 2,3-Hexanedione | Sample roaster | 5 | – | – | 13* | 20 |
| Acetaldehyde | Café grinder | 7 | 238 | 4.9 | 3160 | 0 |
| Acetaldehyde | Ground coffee | 52 | 987 | 10 | 42,631 | 0 |
| Acetaldehyde | Heat sealing bags | 3 | – | – | 42* | 0 |
| Acetaldehyde | Miscellaneous QC | 11 | 57 | 14 | 4,161 | 9.1 |
| Acetaldehyde | Miscellaneous café | 7 | 27 | 14 | 2,012 | 14 |
| Acetaldehyde | Packaging roasted coffee | 18 | 65 | 2.6 | 318 | 0 |
| Acetaldehyde | QC grinding | 9 | 22 | 66 | 20,429 | 22 |
| Acetaldehyde | Roasted coffee | 54 | 16 | 14 | 1,193 | 17 |
| Acetaldehyde | Roasted coffee in bag | 5 | 229 | 28 | 52,991 | 0 |
| Acetaldehyde | Roasted coffee in container | 53 | 305 | 23 | 51,937 | 7.5 |
| Acetaldehyde | Roaster cooling drum | 12 | 22 | 2.3 | 86 | 0 |
| Acetaldehyde | Roaster door | 10 | 25 | 1.7 | 59 | 0 |
| Acetaldehyde | Roasting | 12 | 24 | 15 | 1933 | 17 |
| Acetaldehyde | Sample roaster | 5 | 511 | 7.6 | 13,875 | 0 |
| Acetone | Café grinder | 7 | 168 | 3.5 | 1310 | 14 |
| Acetone | Ground coffee | 52 | 1,645 | 9.3 | 63,968 | 0 |
| Acetone | Heat sealing bags | 3 | – | – | 120* | 0 |
| Acetone | Miscellaneous QC | 11 | 80 | 9.1 | 2963 | 9 |
| Acetone | Miscellaneous café | 7 | 88 | 2.9 | 487 | 0 |
| Acetone | Packaging roasted coffee | 18 | 105 | 3.6 | 851 | 0 |
| Acetone | QC grinding | 9 | 185 | 4.1 | 1,831 | 0 |
| Acetone | Roasted coffee | 54 | 67 | 4.6 | 813 | 2 |
| Acetone | Roasted coffee in bag | 5 | 342 | 43 | 156,084 | 0 |
| Acetone | Roasted coffee in container | 53 | 546 | 12 | 31,654 | 4 |
| Acetone | Roaster cooling drum | 12 | 27 | 14 | 1,981 | 17 |
| Acetone | Roaster door | 10 | 9 | 30 | 2,219 | 30 |
| Acetone | Roasting | 12 | 87 | 4.6 | 1,059 | 0 |
| Acetone | Sample roaster | 5 | – | – | 232* | 40 |
| Acetonitrile | Café grinder | 7 | – | – | 3.6* | 43 |
| Acetonitrile | Ground coffee | 52 | 0.2 | 50 | 116 | 67 |
| Acetonitrile | Heat sealing bags | 3 | – | – | 1.0* | 67 |
| Acetonitrile | Miscellaneous QC | 11 | 0.2 | 11 | 12 | 55 |
| Acetonitrile | Miscellaneous café | 7 | – | – | 2.0* | 57 |
| Acetonitrile | Packaging roasted coffee | 18 | 0.4 | 1.6 | 0.9 | 72 |
| Acetonitrile | QC grinding | 9 | – | – | 6.6* | 67 |
| Acetonitrile | Roasted coffee | 54 | 0.2 | 4.8 | 2.6 | 69 |
| Acetonitrile | Roasted coffee in bag | 5 | – | – | 23* | 40 |
| Acetonitrile | Roasted coffee in container | 53 | 0.1 | 19 | 10 | 72 |
| Acetonitrile | Roaster cooling drum | 12 | – | – | 1.0* | 92 |
| Acetonitrile | Roaster door | 10 | – | – | 1.0* | 80 |
| Acetonitrile | Roasting | 12 | – | – | 0.8* | 75 |
| Acetonitrile | Sample roaster | 5 | – | – | 89* | 80 |
| α-Pinene | Café grinder | 7 | 3.9 | 1.6 | 8.0 | 0 |
| α-Pinene | Ground coffee | 52 | 0.9 | 1.9 | 2.7 | 44 |
| α-Pinene | Heat sealing bags | 3 | – | – | 1.7* | 67 |
| α-Pinene | Miscellaneous QC | 11 | 1.7 | 1.3 | 2.6 | 0 |
| α-Pinene | Miscellaneous café | 7 | 2.9 | 1.5 | 5.8 | 0 |
| α-Pinene | Packaging roasted coffee | 18 | 0.7 | 2.4 | 3.0 | 56 |
| α-Pinene | QC grinding | 9 | 1.6 | 1.5 | 3.2 | 11 |
| α-Pinene | Roasted coffee | 54 | 1.0 | 1.8 | 2.5 | 19 |
| α-Pinene | Roasted coffee in bag | 5 | – | – | 1.4* | 40 |
| α-Pinene | Roasted coffee in container | 53 | 0.9 | 1.8 | 2.3 | 19 |
| α-Pinene | Roaster cooling drum | 12 | 1.0 | 1.7 | 2.4 | 25 |
| α-Pinene | Roaster door | 10 | 1.0 | 1.7 | 2.4 | 10 |
| α-Pinene | Roasting | 12 | 1.0 | 1.5 | 1.9 | 8.3 |
| α-Pinene | Sample roaster | 5 | – | – | 1.9* | 20 |
| Benzene | Café grinder | 7 | 1.0 | 1.8 | 2.6 | 0 |
| Benzene | Ground coffee | 52 | 2.4 | 6.5 | 52 | 19 |
| Benzene | Heat sealing bags | 3 | – | – | 1.2* | 67 |
| Benzene | Miscellaneous QC | 11 | 1.0 | 2.5 | 4.7 | 9.1 |
| Benzene | Miscellaneous café | 7 | 0.5 | 2.2 | 2.0 | 29 |
| Benzene | Packaging roasted coffee | 18 | 0.2 | 7.1 | 4.8 | 67 |
| Benzene | QC grinding | 9 | 1.6 | 2.4 | 6.8 | 11 |
| Benzene | Roasted coffee | 54 | 0.6 | 2.6 | 2.9 | 19 |
| Benzene | Roasted coffee in bag | 5 | – | – | 13* | 80 |
| Benzene | Roasted coffee in container | 53 | 1.8 | 3.5 | 15 | 7.5 |
| Benzene | Roaster cooling drum | 12 | 0.4 | 4.5 | 4.8 | 33 |
| Benzene | Roaster door | 10 | 1.0 | 2.7 | 4.8 | 0 |
| Benzene | Roasting | 12 | 0.5 | 2.0 | 1.6 | 17 |
| Benzene | Sample roaster | 5 | 2.2 | 2.8 | 12 | 0 |
| *d*-Limonene | Café grinder | 7 | 6.4 | 1.4 | 11 | 0 |
| *d*-Limonene | Ground coffee | 52 | 4.1 | 3.3 | 29 | 23 |
| *d*-Limonene | Heat sealing bags | 3 | – | – | 44* | 33 |
| *d*-Limonene | Miscellaneous QC | 11 | 4.7 | 5.7 | 81 | 9.1 |
| *d*-Limonene | Miscellaneous café | 7 | 5.2 | 1.5 | 10 | 0 |
| *d*-Limonene | Packaging roasted coffee | 18 | 5.1 | 5.0 | 73 | 11 |
| *d*-Limonene | QC grinding | 9 | 4.0 | 11 | 197 | 22 |
| *d*-Limonene | Roasted coffee | 54 | 1.1 | 4.8 | 15 | 39 |
| *d*-Limonene | Roasted coffee in bag | 5 | – | – | 3.0* | 80 |
| *d*-Limonene | Roasted coffee in container | 53 | 2.0 | 2.2 | 7.7 | 19 |
| *d*-Limonene | Roaster cooling drum | 12 | 2.0 | 9.5 | 78 | 33 |
| *d*-Limonene | Roaster door | 10 | 3.5 | 5.9 | 64 | 20 |
| *d*-Limonene | Roasting | 12 | 4.5 | 3.5 | 35 | 8.3 |
| *d*-Limonene | Sample roaster | 5 | 2.1 | 1.6 | 4.3 | 0 |
| Ethanol | Café grinder | 7 | 313 | 1.7 | 714 | 14 |
| Ethanol | Ground coffee | 52 | 95 | 5.4 | 1,501 | 1.9 |
| Ethanol | Heat sealing bags | 3 | – | – | 30* | 0 |
| Ethanol | Miscellaneous QC | 11 | 89 | 3.3 | 635 | 0 |
| Ethanol | Miscellaneous café | 7 | 164 | 1.6 | 370 | 0 |
| Ethanol | Packaging roasted coffee | 18 | 30 | 2.0 | 93 | 0 |
| Ethanol | QC grinding | 9 | 72 | 2.9 | 399 | 0 |
| Ethanol | Roasted coffee | 54 | 46 | 6.2 | 915 | 3.7 |
| Ethanol | Roasted coffee in bag | 5 | 181 | 2.5 | 833 | 0 |
| Ethanol | Roasted coffee in container | 53 | 49 | 4.0 | 489 | 1.9 |
| Ethanol | Roaster cooling drum | 12 | 39 | 3.3 | 274 | 0 |
| Ethanol | Roaster door | 10 | 38 | 2.6 | 184 | 0 |
| Ethanol | Roasting | 12 | 17 | 7.9 | 514 | 8.3 |
| Ethanol | Sample roaster | 5 | 112 | 1.3 | 181 | 0 |
| Ethylbenzene | Café grinder | 7 | 1.1 | 1.7 | 2.8 | 0 |
| Ethylbenzene | Ground coffee | 52 | 0.6 | 4.5 | 7.6 | 54 |
| Ethylbenzene | Heat sealing bags | 3 | – | – | 1.7* | 67 |
| Ethylbenzene | Miscellaneous QC | 11 | 1.2 | 2.2 | 4.4 | 18 |
| Ethylbenzene | Miscellaneous café | 7 | – | – | 2.7* | 43 |
| Ethylbenzene | Packaging roasted coffee | 18 | – | – | 2.6* | 89 |
| Ethylbenzene | QC grinding | 9 | 1.2 | 2.5 | 5.5 | 22 |
| Ethylbenzene | Roasted coffee | 54 | 0.5 | 2.2 | 1.8 | 37 |
| Ethylbenzene | Roasted coffee in bag | 5 | – | – | 1.3* | 40 |
| Ethylbenzene | Roasted coffee in container | 53 | 1.1 | 2.0 | 3.2 | 11 |
| Ethylbenzene | Roaster cooling drum | 12 | 0.7 | 4.3 | 7.5 | 42 |
| Ethylbenzene | Roaster door | 10 | 0.7 | 4.1 | 7.3 | 30 |
| Ethylbenzene | Roasting | 12 | 0.3 | 5.1 | 4.9 | 50 |
| Ethylbenzene | Sample roaster | 5 | 2.1 | 1.6 | 4.8 | 0 |
| Isopropyl alcohol | Café grinder | 7 | 6.3 | 3.7 | 53 | 14 |
| Isopropyl alcohol | Ground coffee | 52 | 11 | 21 | 1,590 | 27 |
| Isopropyl alcohol | Heat sealing bags | 3 | – | – | 11* | 0 |
| Isopropyl alcohol | Miscellaneous QC | 11 | 24 | 2.4 | 97 | 0 |
| Isopropyl alcohol | Miscellaneous café | 7 | 7.4 | 1.4 | 13 | 0 |
| Isopropyl alcohol | Packaging roasted coffee | 18 | 60 | 7.5 | 1,641 | 0 |
| Isopropyl alcohol | QC grinding | 9 | 16 | 2.2 | 59 | 0 |
| Isopropyl alcohol | Roasted coffee | 54 | 5.1 | 6.4 | 108 | 22 |
| Isopropyl alcohol | Roasted coffee in bag | 5 | – | – | 24* | 40 |
| Isopropyl alcohol | Roasted coffee in container | 53 | 4.9 | 3.3 | 35 | 15 |
| Isopropyl alcohol | Roaster cooling drum | 12 | 6.1 | 2.2 | 23 | 8.3 |
| Isopropyl alcohol | Roaster door | 10 | 12 | 3.4 | 85 | 0 |
| Isopropyl alcohol | Roasting | 12 | 4.8 | 13 | 312 | 25 |
| Isopropyl alcohol | Sample roaster | 5 | 20 | 2.3 | 75 | 0 |
| *m,p*-Xylene | Café grinder | 7 | 1.8 | 1.6 | 4.0 | 0 |
| *m,p*-Xylene | Ground coffee | 52 | 1.1 | 4.6 | 14 | 37 |
| *m,p*-Xylene | Heat sealing bags | 3 | – | – | 1.9* | 0 |
| *m,p*-Xylene | Miscellaneous QC | 11 | 2.1 | 3.0 | 12 | 0 |
| *m,p*-Xylene | Miscellaneous café | 7 | 1.2 | 2.3 | 4.6 | 14 |
| *m,p*-Xylene | Packaging roasted coffee | 18 | 0.4 | 3.1 | 2.6 | 56 |
| *m,p*-Xylene | QC grinding | 9 | 2.1 | 3.5 | 16 | 11 |
| *m,p*-Xylene | Roasted coffee | 54 | 0.9 | 1.9 | 2.6 | 13 |
| *m,p*-Xylene | Roasted coffee in bag | 5 | – | – | 4.2* | 20 |
| *m,p*-Xylene | Roasted coffee in container | 53 | 1.4 | 2.3 | 5.4 | 9.4 |
| *m,p*-Xylene | Roaster cooling drum | 12 | 1.1 | 5.8 | 20 | 25 |
| *m,p*-Xylene | Roaster door | 10 | 1.1 | 4.4 | 12 | 20 |
| *m,p*-Xylene | Roasting | 12 | 0.7 | 3.0 | 4.4 | 17 |
| *m,p*-Xylene | Sample roaster | 5 | 3.8 | 1.6 | 7.9 | 0 |
| Methyl methacrylate | Café grinder | 7 | – | – | 3.6* | 57 |
| Methyl methacrylate | Ground coffee | 52 | 0.1 | 10 | 4.6 | 75 |
| Methyl methacrylate | Heat sealing bags | 3 | – | – | 1.8* | 67 |
| Methyl methacrylate | Miscellaneous QC | 11 | 0.2 | 39 | 57 | 55 |
| Methyl methacrylate | Miscellaneous café | 7 | – | – | 3.4* | 71 |
| Methyl methacrylate | Packaging roasted coffee | 18 | 0.2 | 5.9 | 3.5 | 67 |
| Methyl methacrylate | QC grinding | 9 | – | – | 6.5* | 56 |
| Methyl methacrylate | Roasted coffee | 54 | 0.2 | 5.3 | 2.6 | 63 |
| Methyl methacrylate | Roasted coffee in bag | 5 | – | – | 3.7* | 80 |
| Methyl methacrylate | Roasted coffee in container | 53 | 0.7 | 4.1 | 6.8 | 30 |
| Methyl methacrylate | Roaster cooling drum | 12 | 0.4 | 5.5 | 6.0 | 50 |
| Methyl methacrylate | Roaster door | 10 | 0.1 | 22 | 19 | 50 |
| Methyl methacrylate | Roasting | 12 | 0.2 | 15 | 12 | 58 |
| Methyl methacrylate | Sample roaster | 5 | – | – | 9.1* | 20 |
| *o*-Xylene | Café grinder | 7 | 0.9 | 1.6 | 1.9 | 0 |
| *o*-Xylene | Ground coffee | 52 | 0.5 | 6.6 | 10 | 54 |
| *o*-Xylene | Heat sealing bags | 3 | – | – | 1.7* | 33 |
| *o*-Xylene | Miscellaneous QC | 11 | 1.1 | 2.3 | 4.2 | 9.1 |
| *o*-Xylene | Miscellaneous café | 7 | 0.6 | 2.0 | 1.9 | 14 |
| *o*-Xylene | Packaging roasted coffee | 18 | – | – | 2.3* | 78 |
| *o*-Xylene | QC grinding | 9 | 1.1 | 2.6 | 5.2 | 11 |
| *o*-Xylene | Roasted coffee | 54 | 0.5 | 1.9 | 1.5 | 24 |
| *o*-Xylene | Roasted coffee in bag | 5 | – | – | 1.1* | 40 |
| *o*-Xylene | Roasted coffee in container | 53 | 0.7 | 2.5 | 2.9 | 21 |
| *o*-Xylene | Roaster cooling drum | 12 | 0.7 | 3.8 | 6.2 | 25 |
| *o*-Xylene | Roaster door | 10 | 0.4 | 6.9 | 8.9 | 40 |
| *o*-Xylene | Roasting | 12 | 0.4 | 3.9 | 3.2 | 42 |
| *o*-Xylene | Sample roaster | 5 | 1.7 | 1.6 | 3.6 | 0 |
| Styrene | Café grinder | 7 | 2.3 | 5.1 | 33 | 29 |
| Styrene | Ground coffee | 52 | 0.3 | 8.0 | 8.9 | 69 |
| Styrene | Heat sealing bags | 3 | – | – | 1.2* | 67 |
| Styrene | Miscellaneous QC | 11 | – | – | 1.9* | 73 |
| Styrene | Miscellaneous café | 7 | 1.2 | 4.4 | 13 | 29 |
| Styrene | Packaging roasted coffee | 18 | – | – | 2.0* | 94 |
| Styrene | QC grinding | 9 | 0.7 | 2.4 | 2.7 | 44 |
| Styrene | Roasted coffee | 54 | 0.3 | 3.5 | 2.6 | 65 |
| Styrene | Roasted coffee in bag | 5 | – | – | 1.7* | 80 |
| Styrene | Roasted coffee in container | 53 | 0.9 | 3.0 | 5.5 | 36 |
| Styrene | Roaster cooling drum | 12 | – | – | 1.2* | 67 |
| Styrene | Roaster door | 10 | – | – | 11* | 80 |
| Styrene | Roasting | 12 | 0.4 | 3.0 | 2.5 | 58 |
| Styrene | Sample roaster | 5 | – | – | 1.0* | 80 |
| Toluene | Café grinder | 7 | 8.7 | 1.9 | 26 | 0 |
| Toluene | Ground coffee | 52 | 5.6 | 4.4 | 63 | 3.8 |
| Toluene | Heat sealing bags | 3 | – | – | 4.8* | 0 |
| Toluene | Miscellaneous QC | 11 | 4.4 | 2.4 | 18 | 0 |
| Toluene | Miscellaneous café | 7 | 4.0 | 2.5 | 18 | 0 |
| Toluene | Packaging roasted coffee | 18 | 2.1 | 3.1 | 13 | 5.6 |
| Toluene | QC grinding | 9 | 4.8 | 2.9 | 28 | 0 |
| Toluene | Roasted coffee | 54 | 1.6 | 2.1 | 5.3 | 1.9 |
| Toluene | Roasted coffee in bag | 5 | – | – | 14* | 20 |
| Toluene | Roasted coffee in container | 53 | 4.3 | 2.5 | 20 | 3.8 |
| Toluene | Roaster cooling drum | 12 | 1.4 | 4.3 | 16 | 17 |
| Toluene | Roaster door | 10 | 3.6 | 2.1 | 12 | 0 |
| Toluene | Roasting | 12 | 2.1 | 3.4 | 16 | 8.3 |
| Toluene | Sample roaster | 5 | 4.8 | 1.4 | 8.2 | 0 |
| **FLAVOR** |  |  |  |  |  |  |
| 2,3-Hexanedione | Flavored coffee | 8 | 3.9 | 5.8 | 69 | 38 |
| 2,3-Hexanedione | Flavoring | 9 | – | – | 596* | 56 |
| 2,3-Hexanedione | Ground coffee | 17 | 7.6 | 4.9 | 103 | 12 |
| 2,3-Hexanedione | Miscellaneous QC | 1 | – | – | 1.5* | 0 |
| 2,3-Hexanedione | Packaging roasted coffee | 16 | 4.0 | 3.4 | 29 | 19 |
| 2,3-Hexanedione | Roasted coffee | 3 | – | – | 167* | 33 |
| Acetaldehyde | Flavored coffee | 8 | 366 | 9.7 | 14,801 | 0 |
| Acetaldehyde | Flavoring | 9 | 919 | 10 | 41,508 | 11 |
| Acetaldehyde | Ground coffee | 17 | 757 | 7.8 | 21,935 | 5.9 |
| Acetaldehyde | Miscellaneous QC | 1 | – | – | 83* | 0 |
| Acetaldehyde | Packaging roasted coffee | 16 | 123 | 5.2 | 1,861 | 6.3 |
| Acetaldehyde | Roasted coffee | 3 | – | – | 10,531* | 33 |
| Acetone | Flavored coffee | 8 | 232 | 19 | 28,278 | 13 |
| Acetone | Flavoring | 9 | 341 | 65 | 301,886 | 22 |
| Acetone | Ground coffee | 17 | 477 | 15 | 38,147 | 12 |
| Acetone | Miscellaneous QC | 1 | – | – | 131* | 0 |
| Acetone | Packaging roasted coffee | 16 | 254 | 2.5 | 1153 | 0 |
| Acetone | Roasted coffee | 3 | – | – | 47,532* | 0 |
| Acetonitrile | Flavored coffee | 8 | – | – | 400* | 88 |
| Acetonitrile | Flavoring | 9 | – | – | – | 100 |
| Acetonitrile | Ground coffee | 17 | – | – | 41* | 88 |
| Acetonitrile | Miscellaneous QC | 1 | – | – | – | 100 |
| Acetonitrile | Packaging roasted coffee | 16 | – | – | – | 100 |
| Acetonitrile | Roasted coffee | 3 | – | – | 130* | 33 |
| α-Pinene | Flavored coffee | 8 | – | – | 19* | 88 |
| α-Pinene | Flavoring | 9 | – | – | 159* | 67 |
| α-Pinene | Ground coffee | 17 | 1.3 | 4.4 | 14 | 53 |
| α-Pinene | Miscellaneous QC | 1 | – | – | – | 100 |
| α-Pinene | Packaging roasted coffee | 16 | 2.3 | 1.9 | 6.8 | 31 |
| α-Pinene | Roasted coffee | 3 | – | – | – | 100 |
| Benzene | Flavored coffee | 8 | – | – | 1,452* | 63 |
| Benzene | Flavoring | 9 | – | – | 47* | 89 |
| Benzene | Ground coffee | 17 | 0.4 | 20 | 55 | 53 |
| Benzene | Miscellaneous QC | 1 | – | – | – | 100 |
| Benzene | Packaging roasted coffee | 16 | – | – | 6.0* | 81 |
| Benzene | Roasted coffee | 3 | – | – | 118* | 33 |
| *d*-Limonene | Flavored coffee | 8 | 6.5 | 24 | 1,114 | 38 |
| *d*-Limonene | Flavoring | 9 | – | – | 364* | 56 |
| *d*-Limonene | Ground coffee | 17 | 2.3 | 4.9 | 32 | 47 |
| *d*-Limonene | Miscellaneous QC | 1 | – | – | 20* | 0 |
| *d*-Limonene | Packaging roasted coffee | 16 | 2.0 | 2.7 | 10 | 31 |
| *d*-Limonene | Roasted coffee | 3 | – | – | 33* | 33 |
| Ethanol | Flavored coffee | 8 | 1076 | 61 | 875,317 | 13 |
| Ethanol | Flavoring | 9 | 54,154 | 6.0 | 1,020,990 | 0 |
| Ethanol | Ground coffee | 17 | 1,709 | 5.7 | 29,529 | 0 |
| Ethanol | Miscellaneous QC | 1 | – | – | 162* | 0 |
| Ethanol | Packaging roasted coffee | 16 | 455 | 2.5 | 2,069 | 0 |
| Ethanol | Roasted coffee | 3 | – | – | 672* | 0 |
| Ethylbenzene | Flavored coffee | 8 | – | – | – | 100 |
| Ethylbenzene | Flavoring | 9 | – | – | – | 100 |
| Ethylbenzene | Ground coffee | 17 | – | – | 1.5* | 94 |
| Ethylbenzene | Miscellaneous QC | 1 | – | – | NA | 100 |
| Ethylbenzene | Packaging roasted coffee | 16 | 0.4 | 2.0 | 1.4 | 63 |
| Ethylbenzene | Roasted coffee | 3 | – | – | 7.5* | 33 |
| Isopropyl alcohol | Flavored coffee | 8 | – | – | 205* | 75 |
| Isopropyl alcohol | Flavoring | 9 | – | – | 184* | 78 |
| Isopropyl alcohol | Ground coffee | 17 | 3.8 | 11 | 187 | 59 |
| Isopropyl alcohol | Miscellaneous QC | 1 | – | – | 66* | 0 |
| Isopropyl alcohol | Packaging roasted coffee | 16 | 15 | 21 | 2,272 | 31 |
| Isopropyl alcohol | Roasted coffee | 3 | – | – | 176* | 67 |
| *m,p*-Xylene | Flavored coffee | 8 | – | – | 0.9* | 88 |
| *m,p*-Xylene | Flavoring | 9 | – | – | 0.7* | 89 |
| *m,p*-Xylene | Ground coffee | 17 | – | – | 7.9* | 77 |
| *m,p*-Xylene | Miscellaneous QC | 1 | – | – | – | 100 |
| *m,p*-Xylene | Packaging roasted coffee | 16 | 0.6 | 2.2 | 2.3 | 44 |
| *m,p*-Xylene | Roasted coffee | 3 | – | – | 6.3* | 33 |
| Methyl methacrylate | Flavored coffee | 8 | – | – | – | 100 |
| Methyl methacrylate | Flavoring | 9 | – | – | 7.1* | 89 |
| Methyl methacrylate | Ground coffee | 17 | – | – | – | 100 |
| Methyl methacrylate | Miscellaneous QC | 1 | – | – | – | 100 |
| Methyl methacrylate | Packaging roasted coffee | 16 | – | – | – | 100 |
| Methyl methacrylate | Roasted coffee | 3 | – | – | 62* | 67 |
| *o*-Xylene | Flavored coffee | 8 | – | – | 7.3* | 75 |
| *o*-Xylene | Flavoring | 9 | – | – | – | 100 |
| *o*-Xylene | Ground coffee | 17 | – | – | 1.4* | 82 |
| *o*-Xylene | Miscellaneous QC | 1 | – | – | – | 100 |
| *o*-Xylene | Packaging roasted coffee | 16 | 0.3 | 4.2 | 3.1 | 56 |
| *o*-Xylene | Roasted coffee | 3 | – | – | 0.5* | 67 |
| Styrene | Flavored coffee | 8 | – | – | 37* | 63 |
| Styrene | Flavoring | 9 | – | – | 37* | 89 |
| Styrene | Ground coffee | 17 | 0.3 | 12 | 19 | 71 |
| Styrene | Miscellaneous QC | 1 | – | – | – | 100 |
| Styrene | Packaging roasted coffee | 16 | – | – | 3.4* | 81 |
| Styrene | Roasted coffee | 3 | – | – | 12* | 33 |
| Toluene | Flavored coffee | 8 | 3.3 | 2.9 | 19 | 38 |
| Toluene | Flavoring | 9 | 7.4 | 6.0 | 135 | 33 |
| Toluene | Ground coffee | 17 | 3.5 | 5.0 | 49 | 12 |
| Toluene | Miscellaneous QC | 1 | – | – | 0.7* | 0 |
| Toluene | Packaging roasted coffee | 16 | 2.1 | 2.4 | 8.8 | 13 |
| Toluene | Roasted coffee | 3 | – | – | 72* | 33 |

Note: Chloroform, n-hexane, and methylene chloride not detected in >75% of samples; N=number of samples; GM=geometric mean; ppb=parts per billion; GSD=geometric standard deviation; P95=95^th^ percentile; %BDL=percent samples below the limit of detection max*=maximum presented when <5 measurements were above the detection limit; – =not enough samples above the detection limit to obtain an estimate or only one sample collected
